# Pulse-based cropping systems for soil health restoration, resources conservation, and nutritional and environmental security in rainfed agroecosystems

**DOI:** 10.3389/fmicb.2022.1041124

**Published:** 2023-02-03

**Authors:** Sandeep Kumar, K. A. Gopinath, Seema Sheoran, Ram Swaroop Meena, Ch. Srinivasarao, Sandeep Bedwal, Chetan Kumar Jangir, Kancheti Mrunalini, Ramdhan Jat, C. S. Praharaj

**Affiliations:** ^1^ICAR-Indian Agricultural Research Institute, Regional Station, Karnal, India; ^2^ICAR-Central Research Institute for Dryland Agriculture, Hyderabad, India; ^3^Department of Agronomy, Institute of Agricultural Sciences, Banaras Hindu University, Varanasi, India; ^4^ICAR-National Academy of Agricultural Research Management, Hyderabad, India; ^5^Department of Soil Science, Chaudhary Charan Singh Haryana Agricultural University, Hisar, India; ^6^National Research Centre on Seed Spices, Ajmer, India; ^7^ICAR-Indian Institute of Pulses Research, Kanpur, India; ^8^Department of Agronomy, Chaudhary Charan Singh Haryana Agricultural University, Hisar, India; ^9^ICAR - Directorate of Groundnut Research, Junagadh, Gujarat, India

**Keywords:** climate change, energy and nutrition, pulse-based cropping system, rainfed agroecosystem, soil-human health, sustainable food systems, system productivity and profitability

## Abstract

Pulses are an important source of energy and protein, essential amino acids, dietary fibers, minerals, and vitamins, and play a significant role in addressing global nutritional security. The global pulse area, production, and average productivity increased from 1961 to 2020 (60 years). Pulses are usually grown under rainfed, highly unstable, and complex production environments, with substantial variability in soil and environmental factors, high year-to-year output variability, and variation in soil moisture. Since the last six decades, there is not much satisfactory improvement in the yield of pulses because of their cultivation in harsh environments, coupled with their continuous ignorance of the farmers and governments in policy planning. As a result, the global food supplies through pulses remained negligible and amounted to merely ~1.0% of the total food supply and 1.2% of the vegan food system. In this situation, protein-rich food is still a question raised at the global level to make a malnutrition-free world. Pulses are a vital component of agricultural biological diversity, essential for tackling climate change, and serve as an energy diet for vegetarians. Pulses can mitigate climate change by reducing the dependence on synthetic fertilizers that artificially introduce nitrogen (N) into the soil. The high demand and manufacture of chemical fertilizers emit greenhouse gases (GHGs), and their overuse can harm the environment. In addition, the increasing demand for the vegetal protein under most global agroecosystems has to be met with under a stressed rainfed situation. The rainfed agroecosystem is a shelter for poor people from a significant part of the globe, such as Africa, South Asia, and Latin America. Nearly, 83% [over 1,260 million hectares (ha)] of cultivated land comes under rainfed agriculture, contributing significantly to global food security by supplying over 60% of the food. In rainfed areas, the limitation of natural resources with the shrinking land, continuous nutrient mining, soil fertility depletion, declining productivity factor, constantly depleting water availability, decreasing soil carbon (C) stock, augmented weed menace, ecological instability, and reduced system productivity are creating a more challenging situation. Pulses, being crops of marginal and semi-marginal soils of arid and semi-arid climates, require less input for cultivation, such as water, nutrients, tillage, labor, and energy. Furthermore, accommodation of the area for the cultivation of pulses reduces the groundwater exploitation, C and N footprints, agrochemical application in the cropping systems, and ill effects of climate change due to their inherent capacity to withstand harsh soil to exhibit phytoremediation properties and to stand well under stressed environmental condition. This article focuses on the role of pulses in ecological services, human wellbeing, soil, environmental health, and economic security for advanced sustainability. Therefore, this study will enhance the understanding of productivity improvement in a system-based approach in a rainfed agroecosystem through the involvement of pulses. Furthermore, the present study highlighted significant research findings and policy support in the direction of exploring the real yield potential of pulses. It will provide a road map to producers, researchers, policymakers, and government planners working on pulses to promote them in rainfed agroecosystems to achieve the United Nations (UN's) Sustainable Development Goals (SDGs).

## 1. Introduction

Approximately 3.1 billion people in the world (~40% of the global population) had experienced hunger or did not have a healthy diet in 2020, and up to 112 million people experienced hunger from 2019. In addition, ~200 million people are also suffering from malnutrition, in particular, due to a lack of dietary protein, vitamin A, and micronutrients such as zinc (Zn), iron (Fe), selenium (Se), and iodine (I) (Lal, 2016a; Sheoran et al., 2022a). Beyond hunger, a growing population had to reduce their food quantity. Pulses, a rich source of these elements, are potential crops for the food and nutritional security of the global population. Pulses are required in a daily diet of 52 g per capita day^−1^ (Barik, 2021). However, their production and consumption are continuously being ignored; the net result is that the average per capita consumption has stagnated at ~19.5 g over the last three decades (FAOSTAT, 2022). This figure dramatically varies across geographical locations, countries, and socioeconomic classes, being maximum in Eastern Africa (44.2 g per capita day^−1^), Southern Asia and the Caribbean (34.0 g per capita day^−1^), and Western Africa and Central America (~30.0 g per capita day^−1^) (FAOSTAT, 2022). Globally, the average daily consumption of pulses has remained almost stagnant for the last three decades except in South Africa, where its consumption had steadily increased from 21 g per capita day^−1^ in 1985 to 29.5 g per capita day^−1^ in 2019 (Rawal and Navarro, 2019; FAOSTAT, 2022). Along with this development, the global average supply of protein from pulses is also low (4.2 g per capita day^−1^), accounting for only 8.4 and 5.0% of the average protein being supplied from vegetal (50.02 g per capita day^−1^) and vegetal plus animal sources (83.2 g per capita day^−1^), respectively (FAOSTAT, 2022).

As regards the current situation, global food security has become one of concern due to a rapid increase in population, climate change, and limited lands available for cultivation; at the same time, interest in proteins from plant sources is also increasing, as opposed to animal protein, for a healthy living. Pulses are a significant component of agricultural systems for their vital contribution to sustaining agricultural growth due to their resource-conserving nature and environment-friendly attribute; the increase in pulse production acts as a panacea to manage the changing climate (Kumar et al., 2022a). Pulses are treasured by smallholder farmers in rainfed agroecosystems who depend on the food they store between harvests. Even though pulses have several nutritional advantages, their area under cultivation accounted for only 12.7% (93.18 Mha) of cereals' (736 Mha) area and 3.0% [89.82 Mt (million tons)] of their production in 2020 (FAOSTAT, 2022). This stat shows the complete ignorance of pulse cultivation by the farmers and governments on the global platform. Pulses are cultivated primarily in rainfed ecosystems and on marginal soils (depleted and degraded soils) and harsh environments that result in a low, highly variable, and unpredictable production and productivity of pulses, compared to cereals (Shukla et al., 2022). Although, throughout the globe, pulses are essential key players in the rainfed agroecosystem, owing to their ability to withstand and perform well under harsh environments, including water, temperature, fertility, and nutritional stress, and their lesser demands on resources (Lal, 2017). Cultivating pulses in rotation with other crops restores soil health and adaptation and mitigates climate changes in the rainfed agroecosystem. They positively impact soil properties, resource utilization, biological nitrogen fixation (BNF), N economy, and production sustainability. These crops also deliver the effect on soil C and N sequestration and increase the productivity of the companion crops growing in rotational sequence. Overall, pulses positively impact dietary energy, soil and human health, and nutritional, environmental, and economic security.

The present study explicitly attempts to analyze the change in pulse production and consumption patterns across the globe and their impact on nutrient intake in terms of energy and protein. Most data (1961–2022) used in this study have been obtained from the Food and Agriculture Organization of the United Nations (FAOSTAT) assessed in 2022 and analyzed to present a valid conclusion. Overall, this review has made an effort to analyze the significance of pulses in food consumption, nutritional security, and soil health restoration *vis-à-vis* environmental sustainability. This article provides a road map to the producers, researchers, policymakers, and government planners to promote pulses in rainfed agroecosystems under the United Nations (UN) umbrella to achieve the Sustainable Development Goals (SDGs). The SDGs have been employed to increase public awareness about the importance of pulses to achieve advanced sustainability (Diet Health Club, 2014; Lal, 2016a).

## 2. Global trends and status of pulse cultivation

**Figure 1:**
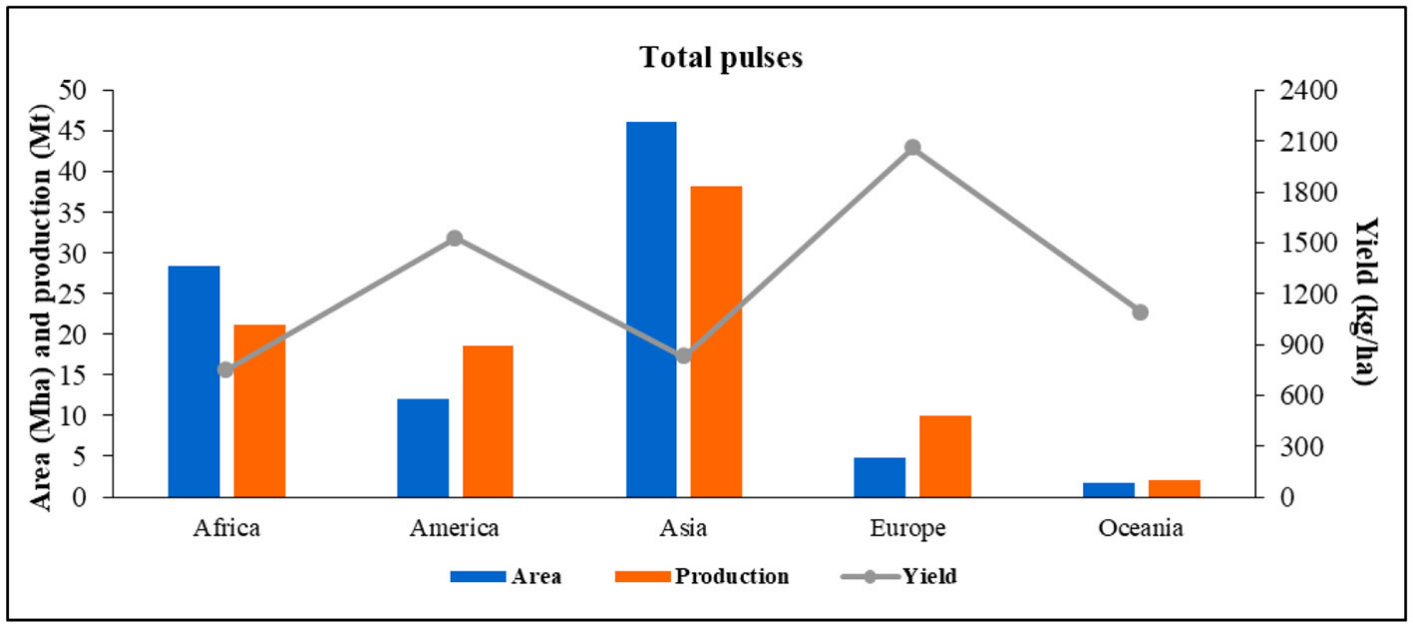
Area, production, and yield of total pulses in different regions of the world in 2020 (Data Sources: FAOSTAT, 2022).

Increasing global pulse production is challenging so as to meet the global demand for pulses. Globally important pulses include dry beans such as *Phaseolus* spp. (kidney bean, pinto bean, black turtle bean, navy bean), mung bean (*Vigna radiata*), urd bean (*Vigna mungo*), rice bean (*Vigna umbellata*), moth bean (*Vigna aconitifolia*), lima bean (*Phaseolus lunatus*), adzuki bean (*Vigna angularis*), scarlet runner bean (*Phaseolus coccineus*), and Tepary bean (*Phaseolus acutifolius*); chickpea (*Cicer arietinum*); dry pea including garden pea (*Pisum sativum* var. *sativum*) and protein pea (*Pisum sativum* var. *arvense*); cowpea (*Vigna unguiculata*); lentil (*Lens culinaris*); pigeon pea (*Cajanus cajan*); broad bean including horse bean (*Vicia faba equina*), broad bean (*Vicia faba*), and field bean (*Vicia faba*); lupin (*Lupinus* spp.); vetches/common bean (*Vicia sativa*); bambara bean/earth pea (*Vigna subterranea*); and pulses such as lablab (*Dolichos lablab* or *Lablab purpureus*), jack bean (*Canavalia ensiformis*), sword bean (*Canavalia gladiata*), winged bean (*Psophocarpus tetragonolobus*), velvet bean (*Mucuna pruriens* var. *utilis*), and yam bean (*Pachyrhizus erosus*) (https://en.wikipedia.org/wiki/Legume). The average global pulse production increased from 40.8 Mt in 1961 to 89.8 Mt in 2020, with an overall increment of 120.2% (0.82 Mt year^−1^) over the base year. The increase in production was augmented primarily due to the expansion of the area under pulse cultivation from 64.0 Mha in 1961 to 93.2 M ha in 2020 (0.49 M ha year^−1^, 45.6%). Furthermore, with the implementation of improved varieties and agricultural practices, productivity had also increased steadily from 637 kg ha^−1^ in 1961 to 964 kg ha^−1^ in 2020 at an annual increase rate of 5.4 kg ha^−1^ (51.3%), contributing to an increase in total pulse production (Figure 1).

**Figure 2:**
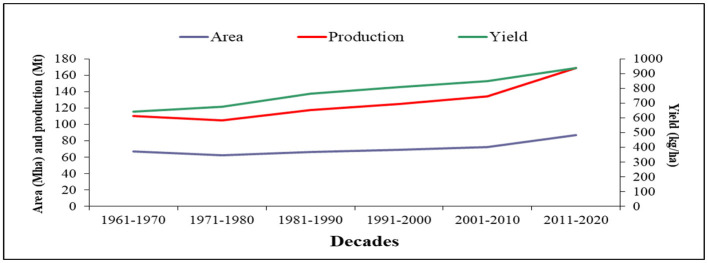
Decadal change in the area, production, and average yield of pulses over the globe (Data sources: FAOSTAT, 2022).

The global area and total production of pulses decreased between the 1960s and the 1980s, but both these parameters increased steadily during the 1990s and the 2000s and increased speedily during the 2010s. In contrast, the average yield of pulses increased consistently from the 1960s to the 2010s (644–964 kg ha^−1^). In comparison to the 1960s, there was a 120.2% increase in production, a 51.3% increase in the average seed yield, and a 45.6% increase in the pulse area until the end of the 2010s (Figure 2).

**Figure 3:**
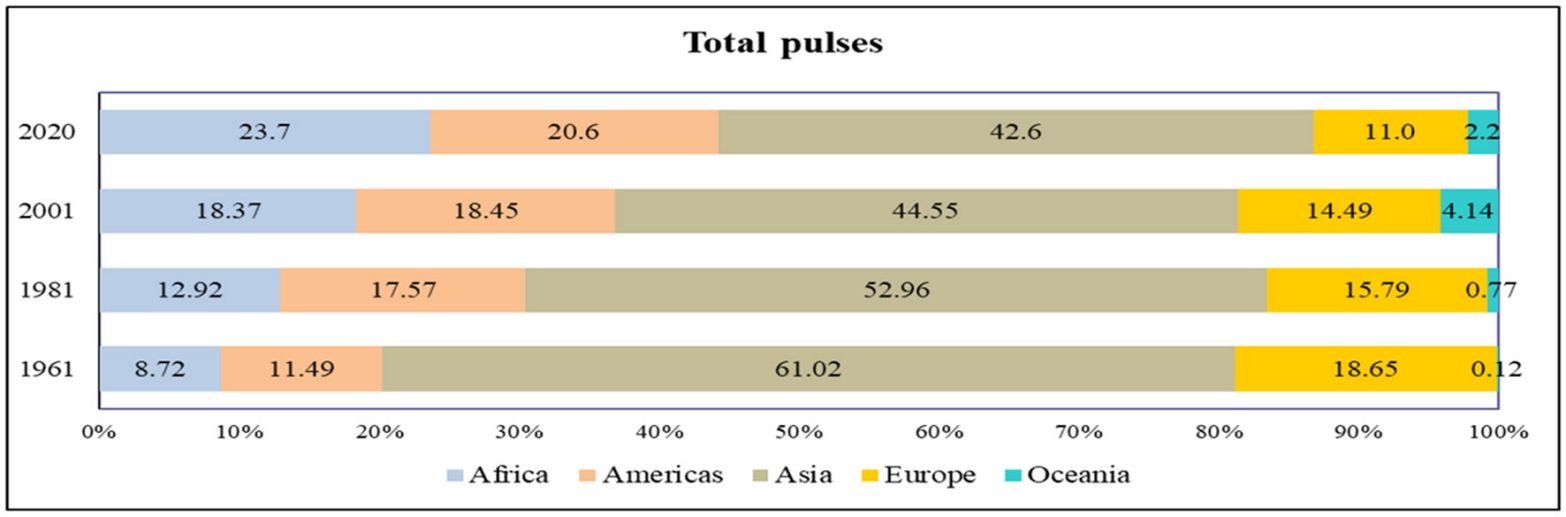
Continent-wise share in the global pulse production, ending 1961, 1981, 2001, and 2020 (in percentage) (Data Sources: FAOSTAT, 2022).

In the long journey of ~60 years (1961–2020), all the continents significantly contributed to improving areas, production, and productivity of pulses (Figure 3).

**Figure 4:**
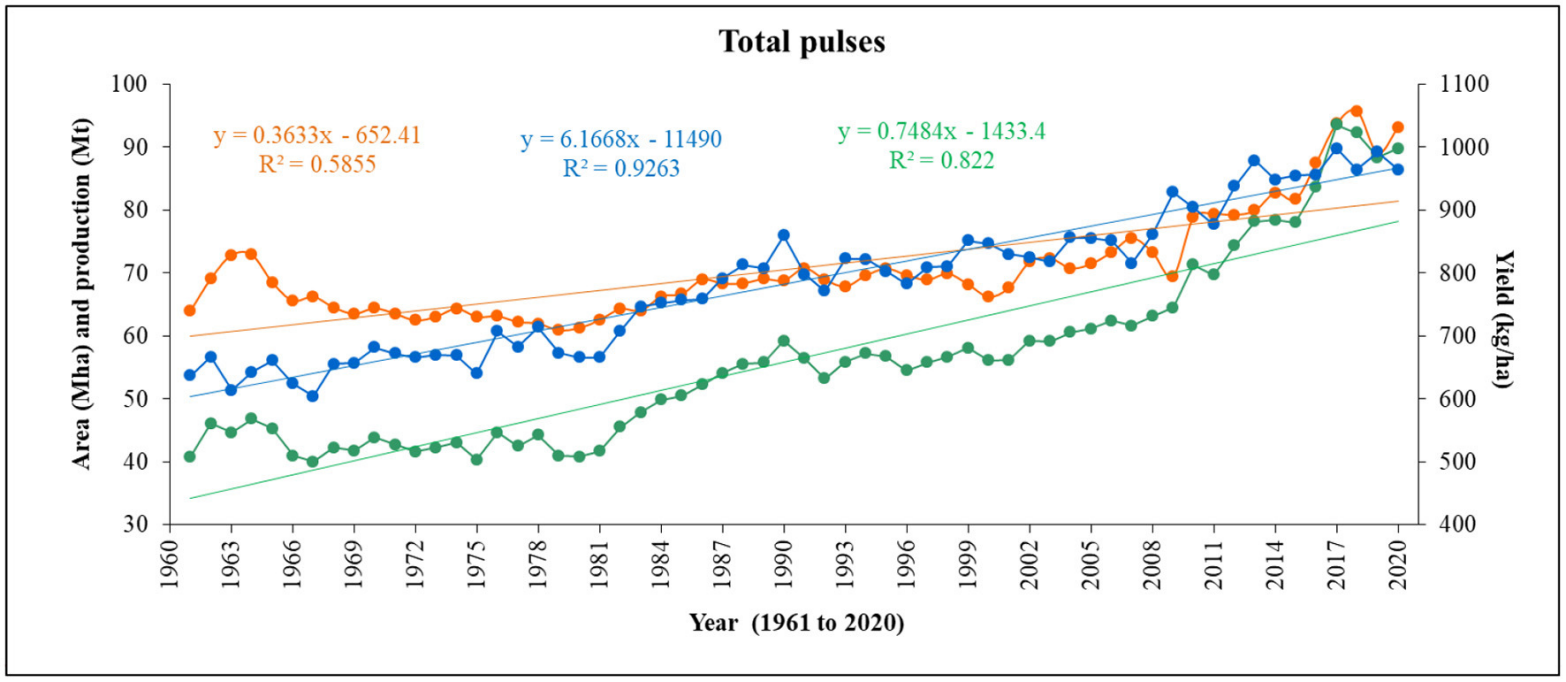
Temporal variability in total area, production, and yield of pulses in the world over the last 60 years (1961–2020) (Data sources: FAOSTAT, 2022).

The analysis of the global pulse data from 1961 to 2020 shows a large temporal and spatial variability in total area, production, and average yields (Figure 4).

As per the data obtained from FAOSTAT (2022), Asia contributed the largest area under pulse cultivation (46.1 Mha), with a maximum production of 38.2 Mt in 2020. Although Asia is the major producer, importer, and consumer of pulses, it contributed 61.0% of the total world's pulse production in 1961; since then, its contribution decreased to 42.6% in 2020 due to a significant increment in areas under pulses in both African (4.0 times) and American (1.8 times) continents. In 2020, Africa, America, and Europe produced 21.2 (23.6%), 18.5 (20.6%), and 9.9 Mt (11.0%) of pulses from 28.4, 12.1, and 4.8 Mha areas, respectively. While Oceania had the least contribution, accounting for only 2.2% of the total production. Productivitywise, the trends in different continents were that Europe had the highest productivity with 2,058 kg ha^−1^, followed by America (1,525 kg ha^−1^), Oceania (1,089 kg ha^−1^), Asia (830 kg ha^−1^), and Africa (749 kg ha^−1^). In 2020, the top pulse-producing countries were India (26.0%), Canada (9.1%), China (5.3%), Myanmar (4.5%), Nigeria (4.1%), Russia (3.8%), and Brazil (3.4%). There are significant interregional and intercountry yield variations. The average yield of developed countries is >1,200 kg ha^−1^ (e.g., Canada and the USA >20 q ha^−1^), while the average yield of developing countries is <1,000 q ha^−1^ (except Ethiopia at 1,880 kg ha^−1^); for instance, most African and South Asian countries' yields are <500 kg ha^−1^ (FAOSTAT, 2022). Still, considering the need to produce more nutritious food, the areas under pulses must increase to 296 Mha by 2030 and 238 Mha by 2100, in addition to the productivity of 1,800 and 2,150 kg ha^−1^, respectively (Lal, 2016a). India is the largest producer (one-fourth), consumer (27%), and importer (14%) of pulses in the world (Dixit et al., 2015; Kaur et al., 2018). It accounts for one-fifth of the total food grain-producing areas of the world and contributes to nearly 7–10% of the total food grain production (Kaur et al., 2018). However, to meet the growing population's domestic demands for food and protein, pulse production needs to increase by 39 Mt by 2050 (Dixit et al., 2015). The major pulses cultivated and consumed in India are chickpea, dry bean (mung bean, urd bean, moth bean, and red kidney bean), pigeon pea, lentils, dry peas, etc., in the order of their decreased total production in 2020.

The data obtained from FAOSTAT (2022) clearly indicate that pulses contribute to the total and vegetal food supplies (Table 1). Yet, globally, the food supply through pulses is negligible, ~1.0% of the total global food supply and 1.2% of the total global vegetal food supply. Of the total food consumption, people in Africa consume the highest pulses, which are 1.9% of the total food consumption and 2.1% of the entire vegetal food system. Whereas the pulse consumption in Oceania is the lowest, it is 0.2 and 0.3% of the continent's total food supply and vegetal food supply, respectively (FAOSTAT, 2022). Of the global vegetal and non-vegetal food supplies, the Asian continent accounts for the maximum shares (61.7 and 54.4%), while Oceania accounts for the minimum shares (0.4 and 0.8%). Asia (57.1%) ranks first in the context of the percentage of the global food supply through pulses, followed by Africa (25.2%), America (14.0%), Europe (3.6%), and Oceania (0.1%) (FAOSTAT, 2022). Among countries with a minimum total food supply of 1.0 Mt, Niger (97.2%) and Afghanistan (96.6%) have the highest share of vegetal food. In contrast, the consumption of non-vegetal food as a part of whole food is the highest in China, Hong Kong SAR (35.1%) (Table 1). Similarly, the contribution of pulses to the total food supply and total vegetal food supply of the country was the highest in Niger (8.6 and 8.9%), closely followed by Ethiopia (5.8 and 6.3%), respectively, which was much higher than the global average (1.0 and 1.2%). The protein supply from pulses, vegetal and non-vegetal sources of the top 10 countries is highlighted in Table 1.

**Table 1:** Dietary habits of the world and the top ten countries with a major share in each item with the minimum total food supply of 1 Mt (FAOSTAT, 2022).

**Vegetal % of total food supply**		**Non-vegetal % of total food supply**		**% share of pulses of total food supply**		**% share of pulses of total vegetal food supply**	
Niger	97.2	CHS	35.1	Niger	8.6	Niger	8.9
Afghanistan	96.6	Albania	28.1	Ethiopia	5.8	Ethiopia	6.3
Nepal	95.3	Austria	24.5	Rwanda	5.3	Burkina Faso	5.8
Benin	94.5	Mongolia	20.1	URT	3.6	Rwanda	5.7
Rwanda	94.3	France	17.0	Burkina Faso	3.5	Guatemala	4.3
Guinea	94.3	Lithuania	16.7	Guatemala	3.5	URT	4.2
Sudan	94.3	Finland	13.8	Haiti	3.4	Haiti	3.9
Tajikistan	93.5	Slovenia	13.4	Kenya	3.2	Cameroon	3.6
Ethiopia	91.9	Indonesia	12.8	Cameroon	3.1	Kenya	3.5
Kenya	91.6	Guatemala	12.6	Togo	2.4	Togo	2.6
World	83.7	World	11.0	World	1.0	World	1.2

CHS, China, Hong Kong SAR; URT, United Republic of Tanzania.

## 3. Pulses for energy intensification and nutritional security

The latest estimates from the United Nations (UN) state that over 3 billion people cannot afford energy food in their daily diet, out of which 57% are residing in Sub-Saharan Africa and southern Asia (FAO, IFAD, UNICEF, WFP, WHO, 2020). According to the data for 2019 assessed from FAOSTAT (August 2022), the vegetal sources that include cereals, starchy roots, sugar crops, sugar and sweeteners, pulses, tree nuts, oil crops, vegetable oils, vegetables, fruits, spices, milk, etc., contribute 83.7% of the total global food supply, being the highest in Africa (89.9%) and the lowest in Oceania (72.2%). Similarly, non-vegetal sources comprising meat, offal, animal fats, eggs, and fish seafood share 11.0% of global food supplies, whereas the supply was the maximum in Oceania (17.1%), followed by the Americas (16.0%) and the least in Africa (5.5%). The remaining food was supplied through stimulants, alcoholic beverages, and other miscellaneous sources.

Globally, the average energy supply through pulses is 67 kcal per capita day^−1^, which in the case of pulses is the highest in Africa (100 kcal per capita day^−1^), is the lowest in Oceania (16 kcal per capita day^−1^) (Table 2), and has an average of 146 kcal per capita day^−1^ in India (Table 2). The same trends follow as regards the quantity of food supply through pulses on a gram (g) capita^−1^ day^−1^ and kilogram (kg) capita^−1^ year^−1^ basis. However, the supply of food, energy, protein, and fat through pulses is much less than that supplied by rice (*Oryza sativa*), wheat (*Triticum aestivum*), and their products globally due to their much higher production and consumption. Nonetheless, pulses are an excellent source of beneficial micronutrients for humans compared to cereals such as rice, wheat, maize (*Zea mays*), and sorghum (*Sorghum bicolor*). In 2019, the global supply of protein through pulses was 4.2 g per capita day^−1^, which was the highest in Africa (6.6 g per capita day^−1^) and the lowest in Oceania (1.04 g per capita day^−1^) (Table 2).

**Table 2:** Food supply through pulses in different regions of the globe, 2019 (FAOSTAT, 2022).

**Element**	**Africa**	**Americas**	**Asia**	**Europe**	**Oceania**
Food supply quantity (000' tons)	13,738	7,646	31,123	1,923	70
Food supply (kcal capita^−1^ day^−1^)	100.2	70.7	64.5	24.1	15.8
Food supply quantity (g capita^−1^ day^−1^)	29.5	20.7	18.6	7.1	4.6
Protein supply quantity (g capita^−1^ day^−1^)	6.6	4.6	3.9	1.6	1.0
Fat supply quantity (g capita^−1^ day^−1^)	0.53	0.36	0.48	0.13	0.09

The average daily intake of protein from pulses and the share of pulses in total protein sources differ among countries. As per FAOSTAT (2022), the average customary per capita protein supply amount through pulses in four countries is more than 20% of the average protein supply amount from all protein sources. These countries include Burundi (45.6%), Rwanda (36.3%), Niger (31.4%), and the United Republic of Tanzania (20.9%) (Table 3).

**Table 3:** Top ten per capita daily protein supplier countries through all sources, vegetal, and pulses (FAOSTAT, 2022).

**Area**	**Value (g)**	**Area**	**Value (g)**	**Area**	**Value (g)**
**All protein sources**		**Vegetal protein**		**Pulse protein**	
Iceland	143.9	Niger	74.4	Niger	26.7
CHS	129.5	Tunisia	72.3	Rwanda	21.4
Israel	126.1	Egypt	71.8	Burundi	19.5
Portugal	117.7	Turkey	71.3	Cameroon	13.8
Finland	117.5	Morocco	71.2	Ethiopia	13.7
Norway	116.3	Burkina Faso	69.3	URT	12.6
Lithuania	116.0	Algeria	66.8	Burkina Faso	11.0
Albania	116.0	Ethiopia	64.2	Comoros	10.8
Denmark	115.4	China	63.5	Cuba	10.5
USA	115.0	Bosnia and Herzegovina	62.7	El Salvador	10.2

CHS, China, Hong Kong SAR; URT, United Republic of Tanzania; USA, United States of America.

The pulse seeds contain a good amount of carbohydrates (40.4–64.2 g) and encompass excellent dietary fiber (10.6–25.6 g) (Table 4). The United States of America's dietary guidelines advocated the daily intake of 33.6 g of dietary fiber for young men and 28.0 g of dietary fiber for young women in the 19–30 age group. The dietary fiber requirement can be fulfilled by consuming 110–320 g of daily pulses, the intake depending on the gender in the same age group and the type of pulse being consumed. A half-cup of pulses (224 g) daily in the diet provides 7–17% dietary fiber, which is 26–68% of the daily recommended fiber intake in women and 18–45% for men (ICRISAT, 2016). One cup (448 g) of cooked chickpea provides 125 g of dietary fiber, accounting for 50% of the daily fiber requirement. In addition, pulses are a beneficial source of protein because they contain amino acids, such as lysine (130 mg g^−1^ in peas to 250 mg g^−1^ in pigeon peas), which complement the amino acid found in cereals (methionine). The pulse protein is cheaper [₹ 100 kg^−1^ protein (US$ 1.0 = ₹ 79.8)] than proteins available in eggs (₹ 200), milk (₹ 300), and fish/meat (₹ 450). It is also easily digestible and ranges from 18.8% in french beans to 36.2% in lupines, with an average value of 20–25% in most pulses (Table 4), and the average is two times that of wheat (~12%) and three times that of rice (6–7%). However, the protein content varies across the varieties of a particular crop, for example, common bean (20.9–29.2%), pea (15.8–32.1%), lentil (19–32%), faba bean (22–36%), chickpea (16–28%), mung bean (21–31%), cowpea (16–31%), and pigeon pea (16–24%) (Burstin et al., 2011).

**Table 4:** Energy profile of common pulses (https://www.nutritionvalue.org).

**Name**	**Carbohydrate (g)**	**Energy (calories)**	**Protein (g)**	**Dietary fiber (g)**	**Fat (g)**
Adzuki bean	62.90	329	19.87	12.7	0.53
Urd bean	58.99	341	25.21	18.3	1.64
Black turtle bean	63.25	339	21.25	15.5	0.90
Borlotti bean/cranberry bean/Roman bean	60.65	335	23.03	24.7	1.23
Broad bean	58.29	341	26.10	25.0	1.53
Chickpea	62.95	378	20.47	12.2	6.04
Cowpea common	60.03	336	23.52	10.6	1.26
French bean	64.11	343	18.81	25.2	2.02
Mung bean	62.62	347	23.86	16.3	1.15
Hyacinth bean	61.52	344	22.94	25.6	1.69
Kidney bean, red	61.29	337	22.53	15.2	1.06
Lentil	63.35	352	24.63	10.7	1.06
Lima bean	63.38	338	21.46	19.0	0.69
Lupine	40.37	371	36.17	18.9	9.74
Moth bean	61.52	343	22.94	0.00	1.61
Navy bean	60.75	337	22.33	15.3	1.50
Pea	61.63	364	23.12	22.2	3.89
Pigeon pea	62.78	343	21.70	15.0	1.49
Pink bean	64.19	343	20.96	12.7	1.13
Pinto bean	62.55	347	21.42	15.5	1.23
White bean	60.27	333	23.36	15.2	0.85

Value for 100 g, raw, mature seeds.

Pulse seeds are a rich source of plant-based protein, dietary fiber, vitamin B, and mineral nutrients such as Fe, Zn, potassium (K), phosphorus (P), calcium (Ca), and magnesium (Mg) (Jangir et al., 2017b). Pulses, contributing ~10% to the daily protein intake and 5% to energy intake, are important for nutritional security. A dietician recommends a daily intake of 0.8 g of protein for every kilogram of bodyweight. Of the total calories required for a vegetarian, ~10% should come from protein. For instance, the daily energy needs for a male having a bodyweight of 80 kg should be 2,640 calories; therefore, his protein requirement is 72 g (80 × 0.90 g kg^−1^). The calories gained from protein can be calculated by multiplying 72 g of protein by four calories g^−1^, which is 288 calories. Thus, ~10.9% of calories are received from protein (288/2,640) (Mangels et al., 2011). So, out of the total daily calorific needs, typically, 10–12% of calories should come from protein, depending on the vegetarian diet, which is 14–18% for non-vegetarians. The pulse seeds are energy-dense with a calorie range of 329 (Adzuki bean) to 372 (chickpea) in 100 g of raw mature seeds. Based on the data in Table 4, a daily intake of 100 g of the raw, mature pulse would provide, on average, 350 calories of energy and 25 g of protein. However, for the daily intake of protein of 30 g per capita, there is a need for 125 g of pulses per day (Lal, 2017).

Pulse seeds are rich in iron (Fe) content, which is 2–16 times those supplied by rice, barley (*Hordeum vulgare*), and maize. For instance, its concentration in pulse seeds varies from 3.40 (french bean) to 10.85 mg (moth bean), higher than those of rice (0.2 mg), wheat (3.52 mg), and maize (0.52 mg) in 100 g of raw, mature product (https://foodstruct.com/). That makes the pulses a potent food for alleviating iron (Fe) deficiency, ranging from 50 to 70% in children and women, particularly in pregnant women. Similarly, the pulses are high in Zn content, varying from 0.95 mg in lima bean to 9.30 mg in hyacinth bean with a typical range of 2–4 mg per 100 g of raw, mature seeds. The zinc (Zn) content in pulses is two times higher than that in maize and three times higher than that in rice grains. Pulses also contain a good amount of manganese (Mn) in their seeds, ranging from 0.52 mg (lima bean) to as high as 21.31 mg in 100 g raw, mature seeds of chickpea. Although, the typical range of Mn content is 1.0 to 2.0 mg 100 g^−1^ seed. In all, a 100 g of cooked chickpea and pigeon pea supplies 52.2 and 25.4% of the daily recommended intake of Mn (ICRISAT, 2016). The potassium (K) content in most of the pulses varies between 1,000 and 1,500 mg per 100 g of raw, mature seeds with a maximum content in white bean (1,795 mg), lima bean (1,724 mg), black turtle bean (1,500 mg), and pink bean (1,464 mg). The K content in seeds of pulses is 2–11 times more than those of rice (250 mg), wheat (435 mg), maize (287 mg), sorghum (363 mg), and barley (280 mg) in 100 g of raw, mature product. Potassium has the opposite effect of sodium (Na) on the body, increasing Na excretion in the kidney, and thus drawing water molecules with it. It is required for bones, muscle function, heart diseases, lowering blood pressure, and allowing blood vessels to relax (Rodan, 2017). Similarly, magnesium (Mg) is important for maintaining bodyweight, in addition to various metabolic syndromes associated with cardiovascular disease. In this line, pulse seeds are rich in Mg content, ranging from 47 mg in lentils to 381 mg in moth beans in 100 g raw, mature seeds. A 100 g of cooked chickpea and pigeon pea seeds provides 14 and 13.2% of the daily recommended intake of Mg, respectively (ICRISAT, 2016). Pulses are also considerably richer in calcium (Ca) than most cereals and contain ~100–200 mg of Ca per 100 g of seeds, being the maximum in white beans (240 mg). Pulses are a perfect and balanced diet, as they are low in Na (5–38 mg), glycemic index, and free from cholesterol and gluten. The low Na concentration in a pulse-based diet helps to stabilize blood pressure, stroke, and heart diseases. These crops are an important source of vitamins, particularly vitamin B_9_, known as folate. Our body needs this vitamin to form the DNA, other genetic materials, and cell division (West et al., 2020). The aforementioned information is presented in Table 5.

**Table 5:** Mineral profile of common pulses (https://www.nutritionvalue.org).

**Name**	**P (mg)**	**K (mg)**	**Ca (mg)**	**Mg (mg)**	**Zn (mg)**	**Fe (mg)**	**Cu (mg)**	**Mn (mg)**	**Na (mg)**
Adzuki bean	381	1,254	66	127	5.04	4.98	1.09	1.73	5
Urd bean	379	983	138	267	3.35	7.57	0.98	1.52	38
Black turtle bean	440	1,500	160	160	2.20	8.70	1.00	1.00	9
Borlotti bean/cranberry bean/Roman bean	372	1,332	127	156	3.63	5.00	0.79	0.92	6
Broad bean	421	1,062	103	190	3.14	6.70	0.82	1.63	13
Chickpea	252	718	57	79	2.76	4.31	0.66	21.31	24
Cowpea common	824	1,112	110	184	3.37	8.27	0.84	1.53	16
French bean	304	1,316	186	188	1.90	3.40	0.44	1.20	18
Mung bean	367	1,246	132	189	2.68	6.74	0.94	1.04	15
Hyacinth bean	372	1,235	130	283	9.30	5.10	1.34	1.57	21
Kidney bean, red	406	1,359	83	138	2.79	6.69	0.70	1.11	12
Lentil	281	677	35	47	3.27	6.51	0.75	1.39	6
Lima bean	385	1,724	81	224	0.95	7.51	0.74	0.52	18
Lupine	440	1,013	176	198	4.75	4.36	1.02	2.38	15
Moth bean	489	1,191	150	381	1.92	10.85	0.67	1.82	30
Navy bean	407	1,185	147	175	3.65	5.49	0.83	1.42	5
Pea	334	852	46	63	3.49	4.73	0.81	1.19	5
Pigeon pea	367	1,392	130	183	2.76	5.23	1.06	1.79	17
Pink bean	415	1,464	130	182	3.60	6.77	0.81	1.38	8
Pinto bean	411	1,393	113	176	2.28	5.07	0.89	1.15	12
White bean	301	1,795	240	190	3.67	10.44	0.98	1.80	16

Value for 100 g, raw, mature seeds.

The pulse seeds are abundant in vitamins containing a significant amount of folate (15–649 μg), thiamine (0.273–1.130 mg), riboflavin (0.091–0.333 mg), niacin (0.479–2.630 mg), pantothenic acid (0.732–2.140 mg), vitamin B_6_ (0.140–0.774 mg), vitamin C (0–6.3 mg), vitamin E (0–0.82 mg), and vitamin K (0–15.9 μg) in 100 g of raw, mature seeds (Table 6). The seeds of pinto bean (6.3 mg), mung bean (4.8 mg), lupins (4.8 mg), french bean (4.6 mg), red kidney bean (4.5 mg), lentil (4.5 mg), moth bean (4.0 mg), and chickpea (4.0 mg) contain a good amount of vitamin C, in which most of the cereals (e.g., rice, wheat, and barley) are lacking. It helps the body to absorb Fe in a better way. For instance, in the absence of vitamin C in chickpea, the chickpea seeds absorbed Fe slightly until vitamin C was added. The concentration of vitamin B_12_ is present more in germinated pulse seeds that start to increase after 48 h of germination and are at their peak after 96 h (Rohatgi et al., 1955). A 100 g of cooked chickpea provides 44.5% folate, 24.5% vitamin E, 10.5% thiamine, 10.3% vitamin B_6_, and 4.3% vitamin K in the daily intake. At the same time, 100 g of cooked pigeon pea seed contributes to the daily recommended intake with 28.7% folate, 13.3% thiamine, 5.4% riboflavin, and 3.7% vitamin B_7_. On average, folic acid (folate) present in pulses is nearly six times that in maize and up to 42 times in non-fortified rice. A good amount of folate in pulse seeds has been found to reduce inflammation by reducing inflammatory factors in our bodies.

**Table 6:** Vitamin profile of common pulses (per 100g) (https://www.nutritionvalue.org).

**Name**	**Thiamine (B_1_) (mg)**	**Riboflavin (B_2_) (mg)**	**Niacin (B_3_) (mg)**	**Pantothenic acid (B_5_) (mg)**	**Vit. B_6_ (mg)**	**Folate (B_9_) (μg)**	**Vit. C (mg)**	**Vit. E (mg)**	**Vit. K (μg)**
Adzuki bean	0.455	0.220	2.630	1.471	0.351	622	0.0	0.00	0.0
Urd bean	0.273	0.254	1.447	0.906	0.281	216	0.0	0.00	0.0
Black turtle bean	0.900	0.193	1.955	0.899	0.286	444	0.0	0.21	5.6
Borlotti bean/cranberry bean/Roman bean	0.747	0.213	1.455	0.748	0.309	604	0.0	0.00	0.0
Broad bean	0.555	0.333	2.832	0.976	0.366	423	1.4	0.05	9.0
Chickpea	0.477	0.212	1.541	1.588	0.535	557	4.0	0.82	9.0
Cowpea common	0.853	0.226	2.075	1.496	0.357	633	1.5	0.39	5.0
French bean	0.535	0.221	2.083	0.789	0.401	399	4.6	0.00	0.0
Mung bean	0.621	0.233	2.251	1.91	0.382	625	4.8	0.51	9.0
Hyacinth bean	1.130	0.136	1.610	1.237	0.155	23	0.0	0.00	0.0
Kidney bean, red	0.608	0.215	2.110	0.780	0.397	394	4.5	0.21	5.6
Lentil	0.873	0.211	2.605	2.140	0.540	479	4.5	0.49	0.0
Lima bean	0.507	0.202	1.537	1.355	0.512	395	0.0	0.72	0.0
Lupine	0.640	0.220	2.190	0.750	0.357	355	4.8	0.00	0.0
Moth bean	0.562	0.091	2.800	1.535	0.366	649	4.0	0.00	0.0
Navy bean	0.775	0.164	2.188	0.744	0.428	364	0.0	0.02	2.5
Pea	0.719	0.244	3.608	0.962	0.140	15	1.8	0.12	15.9
Pigeon pea	0.643	0.187	2.965	1.266	0.283	456	0.0	0.00	0.0
Pink bean	0.772	0.192	1.892	0.997	0.527	463	0.0	0.21	5.7
Pinto bean	0.713	0.212	1.174	0.785	0.774	525	6.3	0.21	5.6
White bean	0.437	0.146	0.479	0.732	0.318	388	0.0	0.21	5.6

Value for 100 g, raw, mature seeds.

In addition to the richness of pulse seeds in protein, mineral nutrition, vitamins, carbohydrates, and dietary energy, they also have several other health benefits. A high fiber-based pulse diet (60–70%) helps in weight loss, maintains bowel health, and lowers the risk of colon diseases, apart from reducing heart-related diseases by stabilizing blood pressure and heart inflammation (Jimenes-Cruz et al., 2003). The pulse seeds also enrich the blood and cure skin diseases and ear inflammation, which acts as a tonic and appetizer (Khan et al., 2021). Thus, the consumption of whole pulse seeds protects us against obesity and the development of diabetes and helps to manage people who have already developed type II diabetes mellitus (T2DM). A reduction of 20–30% in the occurrence of diabetes is found if a person takes 2–3 servings of legumes a day. The dietary fiber present in pulse seeds is slowly digestible and aids in alleviating the blood sugar from spiking and the level of insulin. This dietary fiber lowers bad cholesterol in the body [low-density lipoprotein cholesterol (LDL-C)] by preventing bile salts from reabsorbing in the small intestine. For instance, supplementation with seeds of chickpea, navy bean, and pinto bean lowers the LDL-C and high-density lipoprotein cholesterol (HDL-C) in adult women and adult men, and thus the transportation of all fat molecules in extracellular water around the body (Shutler et al., 2007). Therefore, pulses are a good choice for people having diabetes while enhancing insulin resistance. According to a WHO (World Health Organization) (2008) report, up to 80% of heart attacks, diabetes, and strokes can be prevented by adopting a healthy diet with essential pulses. The presence of soluble fiber prevents the absorption of lipids, fats, and cholesterol in the body and advances cardiovascular health, whereas insoluble fibers reduce gastrointestinal (GI) issues (Rawal and Navarro, 2019). The protein in pulses plays an essential role in reducing cardiovascular disease, owing to lower saturated fats (0.53–9.74 g per 100 g of dry seeds) and high dietary fibers. Winham and Hutchins (2007) advocated that consuming baked beans considerably lowers the cholesterol level in the body and the associated risks of cardiovascular diseases. A recent control study found an inverse association between legumes with endometrial and colon cancers in women. For instance, the seeds of the navy bean are rich in saponin, exhibiting antifungal and antibacterial properties that prevent cancer cell growth. Its seeds are also an outstanding source of *p*-coumaric acid and ferulic acid among the varieties of common beans (Luthria et al., 2006). White bean (navy bean) is a well-known copious plant-based source of phosphatidylserine (a phospholipid constituent of the cell membrane) (Souci et al., 2008). Scientific reports have noted that the pinto bean contains a considerable amount of phytoestrogen coumestrol, exhibiting several health benefits. Some of the critical health benefits of pulses are as follows:

1. Pulses lower the risk of coronary heart disease and cardiovascular disease by 22 and 11%, respectively (Arora, 2019). Pulses also modulate the concentration of glucose, insulin, and homocysteine, and lipid peroxidation in patients with coronary artery disease (Jang et al., 2001).
2. Adzuki bean seeds reduce the triglyceride concentration by inhibiting pancreatic lipase activity (hypertriglyceridemia) (Maruyama et al., 2008).
3. Pulses reduce the risk of endometrial, breast, and colon (colorectal adenoma) cancers (Velie et al., 2005).
4. The consumption of whole seeds of beans controls the glycemic index and helps in weight loss (Jimenes-Cruz et al., 2003).
5. Pulse seeds lower the average body mass index (BMI), waist circumference, waist-to-hip ratio, and the risk of obesity (Williams et al., 2000).
6. Vegetables, including some pulse seeds, reduce the risk of lymphoblastic leukemia (Petridou et al., 2005).
7. Chickpea seeds have tonic, appetizer, stimulant and, aphrodisiac, anthelmintic properties (Khan et al., 2021), which reduce the risk of skin and ear inflammation (Gill, 2019) and serum total and LDL cholesterols (hypertriglyceridemia) (Pittaway et al., 2006).

## 4. Rainfed agroecosystem

The importance of rainfed agroecosystems varies regionally, but these agroecosystems provide the majority of food for poor communities in developing countries. The rainfed agroecosystem is home to the production of most of the millets, pulses, cotton (*Gossypium* spp.), oilseeds, and nearly half of the country's rice, apart from providing support to the production of goat, sheep, and cattle. A rainfed agroecosystem is a shelter for poor people in a significant part of the globe, such as Africa and South Asia. Nearly, 83% (over 1,260 million ha) of the cultivated land is under rainfed agriculture, significantly contributing to global food security by supplying around 60% of the food (FAO, 2008) and generating livelihoods in rural areas while providing food for urban people. In North and East Africa, ~80% of the seasonal crops come under the rainfed agroecosystem, whereas in the Sahelian countries, this figure is over 95% of the total cultivated areas (FAO, 2018). Rainfed agriculture covers ~95% of the entire cultivated land of Sub-Saharan Africa, 90% of Latin America, 87% of Southern America, 65% of East Asia, 60% of South Asia, and 75% of Near East and North Africa (Srinivasarao et al., 2013). As per an estimate, ~61% of Indian farmers depend on rainfed farming for their livelihood, while ~60% of the country's gross cropped area falls under a rainfed agroecosystem, contributing ~43% to the national food basket (Venkateswarlu and Prasad, 2012). By taking the example of the Indian subcontinent, even though the full irrigation potential of rainfed areas is realized, 50% of the net cultivated area remains rainfed. In addition, the productivity of the rainfed agroecosystem is still far from the potential yield, especially in developing countries, which have attained only ~30% of the potential yield. In comparison, countries such as Yemen and Pakistan achieved only ~10% of the potential yield in rainfed areas (Ramirez-Vallejo, 2011), often due to low soil fertility, low nutrient inputs, and limited water availability. The soils of rainfed regions face severe issues of soil degradation, reduced soil fertility, multinutrient deficiency, low soil organic carbon (SOC) stock, low soil water retention capacity, high temperatures, limited rainfall, and biotic stresses (Lehman et al., 2015). The high temperatures coupled with heat waves in these ecoregions accelerate the process of soil erosion, evaporation, transpiration from vegetative surfaces, and oxidation of SOC stocks (Jangir et al., 2017a). In addition, the rainfall variability characterized by rare and high-intensity rainfall or drought, and frequent and more prolonged dry spells break the constant water supply of rainfed crops and thus have become the reason for crop failure in these regions (Ramirez-Vallejo, 2011; Kumar et al., 2016). Again, the high-temperature regime and limited water availability reduce the length of the growing season and, thus, the choice of crops and productivity (Lehman et al., 2015).

Furthermore, rainfed agroecosystems are more vulnerable to the consequences of climate change, including variability and unpredictability of rainfall events and increasing frequency of extreme weather events and, as a result, worse effects on productivity. It is also expected that climate change and other soil constraints could convert ~30–60 Mha of cultivated lands in rainfed areas to lands unfit for rainfed agriculture in Sub-Saharan Africa by the 2080s (Fischer et al., 2005). As a result, the extent of soil loss in these regions could range from 5 to 150 Mg ha^−1^ year^−1^ depending upon the soil type, presence of vegetation, and slope gradient (Srinivasarao et al., 2013). This condition can be avoided if the soil is sustainably utilized to reduce the loss of its properties and the impact of climate change. Therefore, there is a lot of scope for sustainable utilization of a potential rainfed ecosystem for enough food production under the scenario of a degrading environment in the future.

A fundamental principle for the sustainability of the rainfed agroecosystem is crop intensification, which can alleviate the existing constraints, adapt well, and produce an optimal yield (Lehman et al., 2015). Pulses can naturally tolerate high temperature and drought stress, owing to their inherently low water requirement. Therefore, pulses, either as monocrop, inter-/mixed-crop, or a sequential crop in rotation in a rainfed agroecosystem, can utilize effectively the available water through their deeper root system (Liu et al., 2019). In addition, these crops conserve the limitedly available soil moisture by providing an efficient ground cover and reducing the risk of water loss through evaporation, leaching, percolation, runoff, soil salinization, nutrient leaching, and soil erosion. In addition, this group of crops produces nutrient-rich biomass that potentially increases SOC levels and contributes to a healthy soil biological community (Lehman et al., 2015). Pulses also fit well in the low-fertile rainfed areas because of their low nutrient requirement coupled with the capacity of BNF for their own use and also leaving some residual N for the use of companion crop in inter-/mixed-crop or subsequent crop in a rotation system (Sheoran et al., 2021). Similarly, these low-fertile rainfed areas are good for restoring soil health, improving micronutrient concentration in the edible parts, and reducing the N and C footprints of the agroecosystems (Lal, 2016b). It is envisaged further that pulse cultivation in a rainfed environment could usher in the second green revolution in the era of emerging health crises such as coronavirus 2019 (COVID-19), climate change, and degrading natural resources for ensuring food and nutritional security, strengthening ecosystem services, and enhancing production sustainability. Therefore, looking toward the miracle of pulses is yet to be harnessed appropriately by focusing more on sustainable pulse production in rainfed conditions by increasing cultivable areas following the best management practices under site-specific soil and environmental conditions.

The area under pulses can be expanded by eliminating fallow periods in rainfed agroecosystems (Ghosh et al., 2019). This step includes the diversification of rice–wheat, maize–wheat, and rice–rice rotation, rice–fallow ecology (22.2 Mha) in India, Bangladesh, and Nepal, and summer fallow (2–3 Mha) in cereal–cereal rotation (rice–wheat in particular) in Indo-Gangetic Plains (IGPs). In addition, these crops can be grown as inter- and/or mixed-crops (e.g., 4–6 Mha in India) in long-duration crops such as banana (*Musa* spp.), sugarcane (*Saccharum officinarum*), newly established orchards, cotton, and millets; relay (*utera/paira*) cultivation in the rice-based system; and relay their inclusion in the agroforestry system. Northeastern hill zones of India under low-input farming could also be a targeted niche for pulse cultivation. Vertically, adequate pulse availability can be ensured by increasing productivity and avoiding postharvest losses. Including pulses in rotation with non-legume crops improves soil health, restores degraded lands, and thus increases the productivity of subsequent crops in rotation and component crops in inter-/mixed-cropping (Meena et al., 2022). Pulses fix atmospheric N at an estimated annual rate of around 3.0 Tg (Teragram = million metric tons). Hence, there is an urgent need to focus more on promoting pulses in the rainfed agroecosystem.

## 5. Pulse-based agroecosystems for rainfed areas

Pulses are an integral part of a profitable agroecosystem because of their energy-rich nutritious food, feed, and forage (Singh et al., 2009). Crops and cropping sequences are chosen for sustainable intensification (SI), economic profitability, energy-rich diversified nutritious food, climate resilience, and ecological services in a system-based approach. This group of food crops can be included in rainfed agroecosystems, owing to their inherent hardy nature, low input requirements, and immense capacity to grow well under prevailing fragile and harsh climatic stresses. Pulses can be grown as green manuring crops, catch crops, and ratoon crops under sequential cropping or monocropping in diverse agroecological regions. In the intercropping system, pulses are taken as a mixed intercrop (e.g., homesteads), row intercrop (e.g., pearl millet + mung bean), and relay intercrop (e.g., mung bean–maize–potato–wheat). The dominant agroecosystem in which pulses are encompassed is double and triple cropping. Relay cropping (*paira/utera*) is practiced in humid regions of northeastern India and drier areas of central and coastal southern India for better use of residual soil moisture. Pulses are the best crop in relay croppings, such as mung bean, urd bean, and lentil, along with rice which facilitates double cropping and system sustainability. In northern India, the development of early maturing cultivars of mung bean, urd bean, and pigeon pea made crop diversification possible and the SI of existing non-pulse crops. In addition to providing excellent nutritious food on hilly slopes, fodder pulses, such as mung bean, urd bean, french bean, and rice bean, also act as tremendous cover crops. Many traditional rainfed agroecosystems have been either modified or replaced by several new pulse-based agroecosystems following sustainability concern and marketing opportunities in rainfed areas. For example, rice–chickpea/lentil, rice–mung bean/urd bean, groundnut (*Arachis hypogaea*)/soybean (*Glycine max*) + pigeon pea, and potato + french bean. In the existing scenario of natural resource degradation in agriculture coupled with increasing demands for nutritious food, the importance of pulses in the agroecosystem is enhanced. Hence, pulses must be incorporated into the predominant rainfed cropping systems. However, there is hardly any scope for expansion of the area under pulses because of the intense competition with cereals and cash crops. Nonetheless, almost 4–6 million ha can be easily devoted to pulses by intercropping them with other crops (Praharaj and Blaise, 2016). This strategy is also in accord with SI and other innovative systems. Some of the possible intercropping systems recommended for the Indian subcontinent are presented in Table 7 (Praharaj and Blaise, 2016; Singh, 2018).

**Table 7:** Possible intercropping systems capable of large-scale advancement and adoption in India.

**Intercropping systems**	**Possible niches**
Cotton + pigeon pea	Madhya Pradesh, Gujarat, Maharashtra, Karnataka, Andhra Pradesh, and Telangana
Cotton + mung bean/urd bean/cowpea	Punjab, Haryana, Madhya Pradesh, Gujarat, Maharashtra, Karnataka, Andhra Pradesh, and Telangana
Indian mustard + chickpea/lentil/mung bean/urd bean	Punjab, Haryana, Uttar Pradesh, Madhya Pradesh, and West Bengal
Potato + French bean	Punjab, Uttar Pradesh, Madhya Pradesh, Bihar, Jharkhand, and West Bengal
Sugarcane + mung bean/urd bean/cowpea	East Uttar Pradesh, Bihar, West Bengal, Maharashtra, Karnataka, Andhra Pradesh, Telangana, and Tamil Nadu
Chickpea/lentil + autumn planted/ratoon sugarcane	Uttar Pradesh, Bihar, and Maharashtra
Soybean + pigeon pea/urd bean	Madhya Pradesh and Maharashtra
Pearl millet/sorghum + pigeon pea	Uttar Pradesh, Madhya Pradesh, Gujarat, Maharashtra, Karnataka, Andhra Pradesh, and Telangana
Groundnut + pigeon pea	Gujarat, Madhya Pradesh, Maharashtra and Uttar Pradesh
Groundnut/sorghum/pearl millet + mung bean/urd bean/cowpea	Rajasthan, Gujarat, Uttar Pradesh, Bihar, Madhya Pradesh, Maharashtra, Karnataka, and Telangana

### 5.1. Significance of pulses in cropping systems in a rainfed agroecosystem

Pulses are vital to food and nutritional security and occupy a unique niche, particularly in dryland or rainfed farming, short growing seasons, summer fallowing, short monsoon season, marginal and/or degraded soils, and impoverished ecoregions (Lal, 2017). In 1961, pulses were grown in 150 countries, and the global number increased to 171 in 2019 (FAOSTAT, 2022). In South Asia, pulses are grown as inter-/mixed crop, sequential crop, monocrop, relay crop, etc., fulfilling the local demands of nutritional food and cash (Adarsh et al., 2019). In Europe, pulses such as faba bean are intercropped with wheat, and the bean supply N to companion cereals. In some regions such as India's Indo-Gangetic plains (IGPs) and the Great Plains of North America, the traditional summer following is being replaced with short-duration pulses (Gan et al., 2015). In Australia, pulses have become an important cash crop since the 1980s, wherein faba beans are grown from 20 to 40°S in the summer season, and chickpeas from 10 to 40°S (south) in the winter season (Lal, 2017). In the African continent, pulses have been grown since ancient times and will continue to be a part of their rainfed agricultural systems that are prone to water scarcity.

Pulses in cereal-based cropping systems have an essential role in improving system productivity, soil fertility status, and soil physical properties by reducing continuous submergence and breaking the chain of insects and pests. Pulses in rotation account for the sustainability of the cropping system by fixing atmospheric N and supplying fixed N to the succeeding cereal. This helps in scavenging mineral N, promoting nutrient cycling deep in the soil, improving the SOC stock, reducing soil compaction and erosion, enhancing weed suppression, and improving mycorrhizal colonization (Jakhar et al., 2017). This crop group enhances employment generation, provides nutrient-rich balanced food, and improves the socioeconomic conditions of small and marginal farmers. Pulse–cereal production system has a low C and water footprint, high soil C accretion capacity, nitrogen fertilization, and improved soil biodiversity (Adarsh et al., 2019). In sequential cropping, component crops compete for residual moisture, nutrient, soil, and light. In the cereal–pulse sequence, the previous pulse crop efficiently utilizes light due to the quick land-covering capacity of the dense canopy (Singh et al., 2009), while the subsequent cereal crop effectively utilizes the residual nutrients and moisture from the previous pulse crop (Adarsh et al., 2019). The improved yield of succeeding cereal crops after pulses results from integrated effects of residual N, soil health improvement, soil water conservation, and pest control in a rainfed agroecosystem.

Pulses aid in economizing the N use in successive non-legume crops, owing to their residual effect. Pulse residues encompass ~20–80 kg N ha^−1^ accounting for nearly 70% of biologically fixed N depending on crop and environmental conditions (Giller, 2001). On average, in sequential cropping, the previous pulse crop supplies 18 to 70 kg N ha^−1^ to the soil (Kaur et al., 2018). This N use remains available for following non-legume crops and, thus, reduces fertilizer requirement up to the extent of 25–30% (Garg and Geetanjali, 2007)—for instance, growing cowpea and mung bean before pearl millet in the preceding season supplies ~60 kg N to the pearl millet crop (Ghosh et al., 2007). While in pulse–wheat sequential cropping, pulses contribute to between 20 and 40% of the N requirement of wheat. However, it is not easy to quantify the amount of fixed N transferred to the subsequent cereal crop due to higher variability in N fixation. The BNF also reduces chemical N inputs in the rotation, which enhances fertilizer use and consequently reduces the environmental consequences of chemical fertilization.

**Figure 5:**
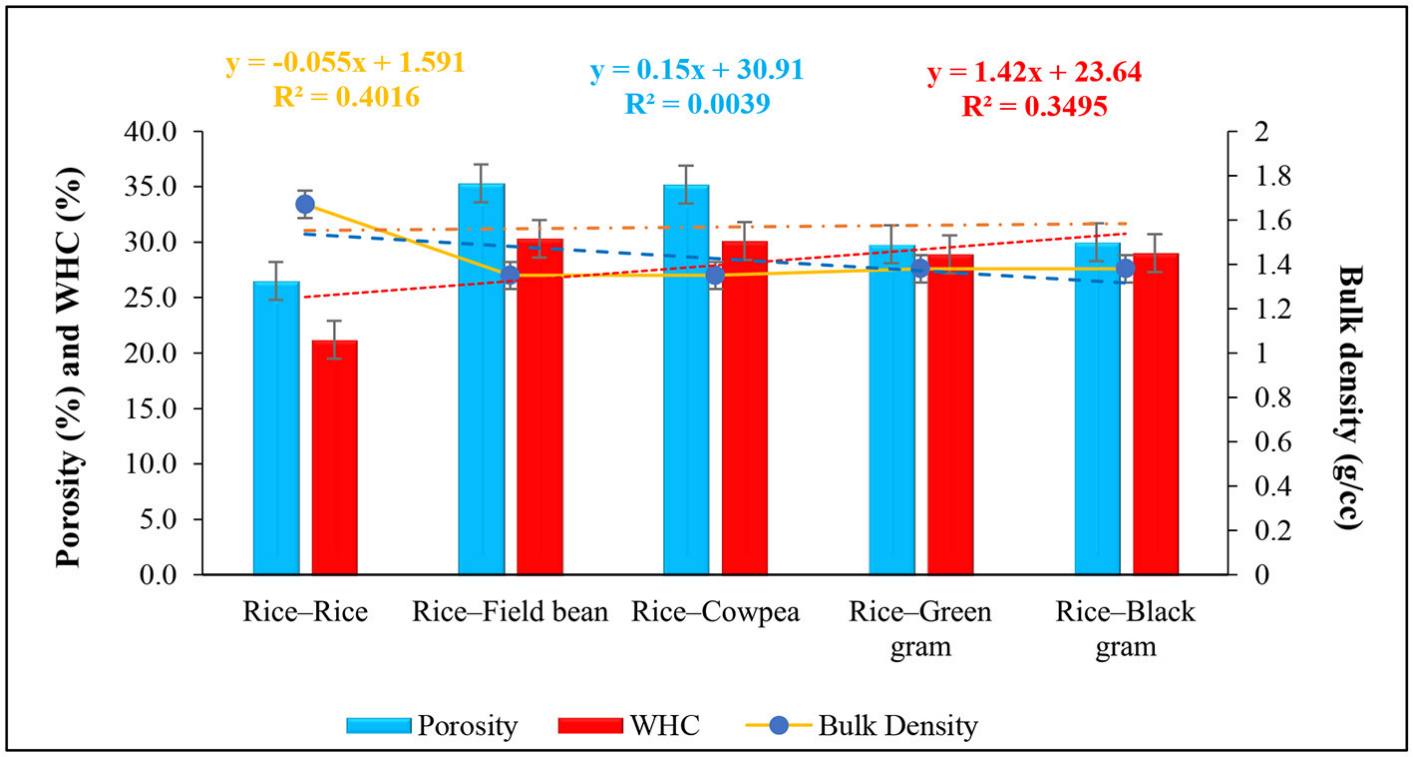
Inclusion of pulses in rotation significantly improved soil porosity (*y* = 0.15*x* + 30.91; *R*^2^ = *0.0039*), water holding capacity (*y* = 1.42*x* + 23.64; *R*^2^ = *0.3495*), and reduced bulk density (*y* = −0.055*x* + 1.591; *R*^2^ = *0.4016*). The rice–field bean (33.21, 42.92, and −19.16%), rice–cowpea (32.83, 41.98, and −19.16%), rice–mung bean (12.45, 36.32, and −17.37%), and rice–urd bean (13.21, 36.79, and −17.37%) improved porosity, WHC and bulk density, respectively over the mono-cropping of rice–rice (*Date source*: Prakash et al., 2008).

Pulses in the cropping system also improve physical soil conditions, such as aggregate stability and soil structure, while reducing bulk density. Aggregate stability is an important index of long-term soil quality improvement in a pulse-based cropping system due to the presence of fungi. The fungi secrete glomalin (a glycoprotein) that entraps solid-oxide-oxygen-ion conducting membrane (SOM), soil minerals, and plant debris to form a stable soil aggregate and improve soil structure further (Singh et al., 2009). In addition, soil bulk density also responds positively to changes in management over the long-term pulse-based cropping system (Figure 5) (Wang et al., 2020). Legumes also enhance earthworm activity, which along with the root channel of pulses, increases soil porosity, promotes aeration, increases water-holding capacity (WHC), and percolates deeper into the subsoil (Kumar et al., 2020) (Figure 5). Pulses influence strongly the exploitation of groundwater. For example, replacing lowland rice with pigeon peas in the rice–wheat system reduces the depletion rate of the groundwater aquifer, improves water use efficiency (WUE), and thus leads to efficient water utilization in the era of climate change accelerated water crises.

**Figure 6:**
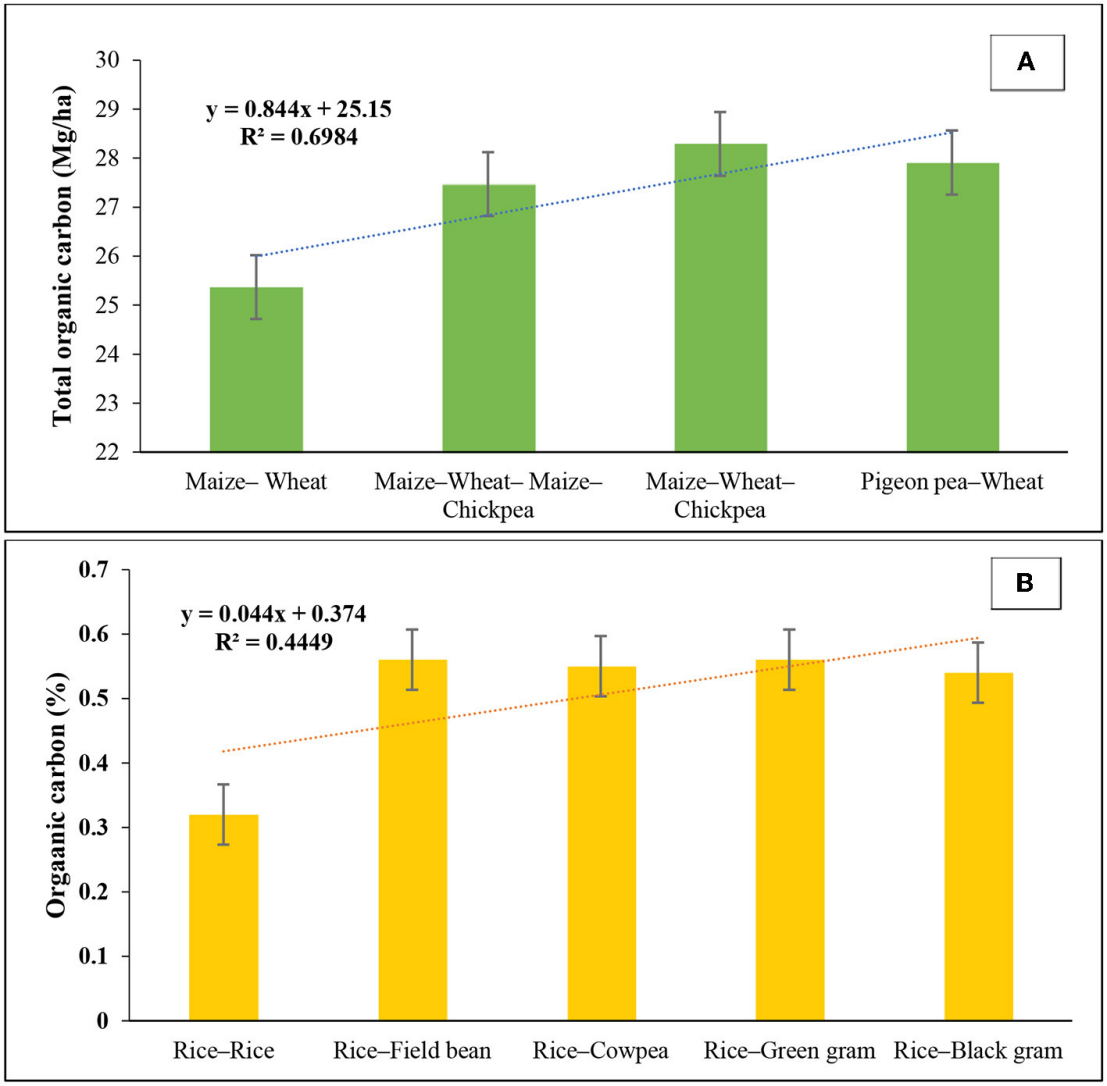
Effect of pulse-based cropping systems on SOC content. The pulse-based cropping systems have a higher soil C sequestration capacity than mono-cropping systems, **(A)** Cropping system of maize–wheat–maize–chickpea (27.5 Mg ha^−1^), maize–wheat–chickpea (28.3 Mg ha^−1^), and pigeon pea–wheat (27.9 Mg ha^−1^) improved SOC by 8.3, 11.5, and 10.0% over the maize–wheat system (25.4 Mg ha^−1^) (*y* = 0.844*x* + 25.15; *R*^2^ = *0.6984*) (*Date source*: Venkatesh et al., 2013); **(B)** The rice–field bean, rice–cowpea, rice–mung bean, and rice–urd bean added 75.0, 71.9, 75.0, and 68.8% more SOC to the soil over rice–rice monoculture (*y* = 0.044*x* + 0.374; *R*^2^ = *0.4449*) (*Data source*: Prakash et al., 2008).

Growing pulses also impacts the chemical properties of the soil and improves soil health status in the cereal-based system (Wang et al., 2020). Pulses fix atmospheric N and reduce the dependency on soil N and thus lower soil pH (Singh et al., 2009). Even a slight decrease in soil pH increases the availability of micronutrients and microbiota activity in the rhizosphere. The capacity of pulses to reduce soil pH is in the decreasing order of chickpeas, peas, and pigeon peas. The decline in soil pH is more beneficial to the following non-pulse crops that are grown under a neutral or saline soil environment. Pulses also add considerable biomass-C to the soil as leaf litter, root biomass, and release of other C compounds (Figures 6A, B). Thus, soil C stock is improved because of the combined effect of eliminating the fallow through the cultivation of a pulse crop and input of extra C to the soil through a pulse-inclusive system (Ghosh et al., 2019). The biomass-N added through these residues becomes available to the successive cereal crops upon decomposition.

Along with N in leaf, shoot, and seed, a large amount of fixed N remains stored in roots and nodules after the crop harvest, which accounts for ~50% of cumulative N in chickpeas and ~30% of cumulative N in lentils and peas (Herridge et al., 2008). In rotation, pulses produce lesser but higher-quality biomass than cereals. They also aid in more biomass production of subsequent non-pulse crops through N benefits that positively impact soil productivity (Liu et al., 2019). Along with N economization in the agroecosystem, the green manuring of pulse-based residues (e.g., mung bean) improves available soil P as the root exudates help to mobilize the soluble P in soil. Roots of pulses are known to release certain acidic substances and H^+^ (hydrogen) ions into the rhizosphere, which solubilize the native insoluble P and thus ensure its availability to the plants. For instance, the secretion of citric acid from roots acidifies the rhizosphere zone that solubilizes P from Ca–P complexes.

Pulse-based rotations are also known to improve the soil's biological environment by releasing low-molecular-weight organic substances *via* root exudates. Soil microbes use these compounds as a source of energy and multiply quickly. The results of a field experiment revealed a more significant soil microbial population and more biomass in the rice–wheat–mung bean and maize–wheat–mung bean systems, compared to those in the rice–wheat and maize–wheat rotation systems, respectively (Kushwaha et al., 2007-2008). The increased microbial population and their functionaries speed up further the process of mineralization–mobilization following the soil environment. The microbial, enzymatic activities and biochemical processes also increase upon soil incorporation of residues of pulses, which over the long term improve soil health and overall system productivity.

Cereal + pulse intercropping is identified as the best intercropping system in the rainfed agroecosystem that helps improve soil health, eliminates soil sickness resulting from monocropping, breaks insect–pest and disease cycles, and has a smothering effect on weeds (Mobasser et al., 2014). Pulses while intercropping also minimize or reduce environmental issues through effective utilization of available resources (i.e., water, nutrient, land, and energy) and fulfill the needs of diverse, balanced food and nutrition needs for poor and marginal farmers. The cereal + pulse intercropping systems under rainfed conditions provide the cereal component's full yield, in addition to the bonus yield of up to 60% of the pulse crop (Ghosh et al., 2007). From a competition point of view in the intercropping system, component crops strive for different resources due to their distinct growth and rooting pattern. For instance, in maize + mung bean intercropping, there is minimal competition for resources because of their differences in peak resource requirement (Adarsh et al., 2019). Similarly, maize + pigeon pea is one of the best examples of minimum competition among component crops. Maize would have already completed its life cycle by the time pigeon pea needs the maximum nutrients at flowering. Thus, the entire system is benefitted through a higher weed smothering efficiency and reduction in the infestation of insect pests and diseases, apart from improving most yield traits due to synergetic interaction. Pulses significantly reduce the N requirement of the component crop and thus decrease external chemical fertilization and associated soil toxification due to chemical leaching in deeper soil strata. In an experiment (Layek et al., 2018) on the intercropping of maize with legume crops, the maize crop responded to up to 60 kg N ha^−1^ application. In contrast, the sole maize responded to a dose of 120 kg N ha^−1^ because the component legume significantly contributed to the N needs of maize. Therefore, pulse-based rotations are needed to sustainably intensify the existing cereal-based production system toward a pulse-based production system in rainfed environments.

### 5.2. Sustainable intensification through a pulse-based rainfed agroecosystem

A continuous increase in food demands for the escalating global population under climate change puts significant pressure on the availability and quality of natural resources (Kumar et al., 2016). Resource constraints over the soil, water, and biodiversity will also directly affect agricultural production in a rainfed agroecosystem in the coming time (Pretty and Bharucha, 2014). At the same time, the rice–wheat system—a significant food production system in South Asia is a supplier of nearly 40% of India's total food production (Gupta and Seth, 2007), causing higher exploitation of natural resources, including groundwater and soil fertility. The yield of the cereal-based cropping system is also either declining or stagnant, which may create problems in achieving the goal of 60% more food production to feed the projected population of 9.1 billion by 2050 (FAO, 2017). The perils of severe water shortage and its deteriorating quality, soil sickness, dwindling economic profitability, and deteriorating available natural resources are some of the barriers to the sustainability of the agroecosystem in South Asia (Chaudhury et al., 2016; Gupta and Kumar, 2019). Therefore, global farmers are challenged to produce more with limited natural resources under adverse conditions.

Food security and production sustainability can be achieved by more efficient input use through desirable modifications to the existing agroecosystem and input management, seeking to capitalize on a more efficient natural resource base (Kumar et al., 2017c; Rani et al., 2020). The SI is an agroecosystem production process wherein crop production is improved without adversely impacting the environment, degradation of natural resources, and cultivation of more land (Pretty and Bharucha, 2014). This implies the use of SI in attaining more food production, advanced environmental goals, and services by various means. The SI seeks to shift toward natural services and social capital without trading off crop productivity and environmental safety. A better concept of SI focuses on the intensification of available resources, making their better use, *for example*, water, land, biodiversity, soil fertility, and technologies (Pretty and Bharucha, 2014). Along with achieving adequate crop production, SI also takes care of ecosystem services and natural resources to maintain productivity factors and mitigate the consequences of the green revolution (Hazra and Bohra, 2020).

For the sake of the sustainability of the agroecosystem, there is a need to formulate and implement some alternative strategies that produce enough diversified and balanced food for the global population and feed to soil with safeguarding the production environment. Pulses are the most realistic and potential crops for SI as these are more eco-friendly and efficient resource utilizers that may become a means to improve system productivity and higher economic returns for the small and marginal farmers in the rainfed ecosystem (Jangir et al., 2016). As these are low-input-requiring crops, they can be grown widely on marginal and wastelands of rainfed areas with fewer management practices and limited water, labor, and other inputs (Adarsh et al., 2019). Recently, the concern about expanding acreage under pulses is growing to progressive niches to achieve self-sufficiency in pulse production.

**Figure 7:**
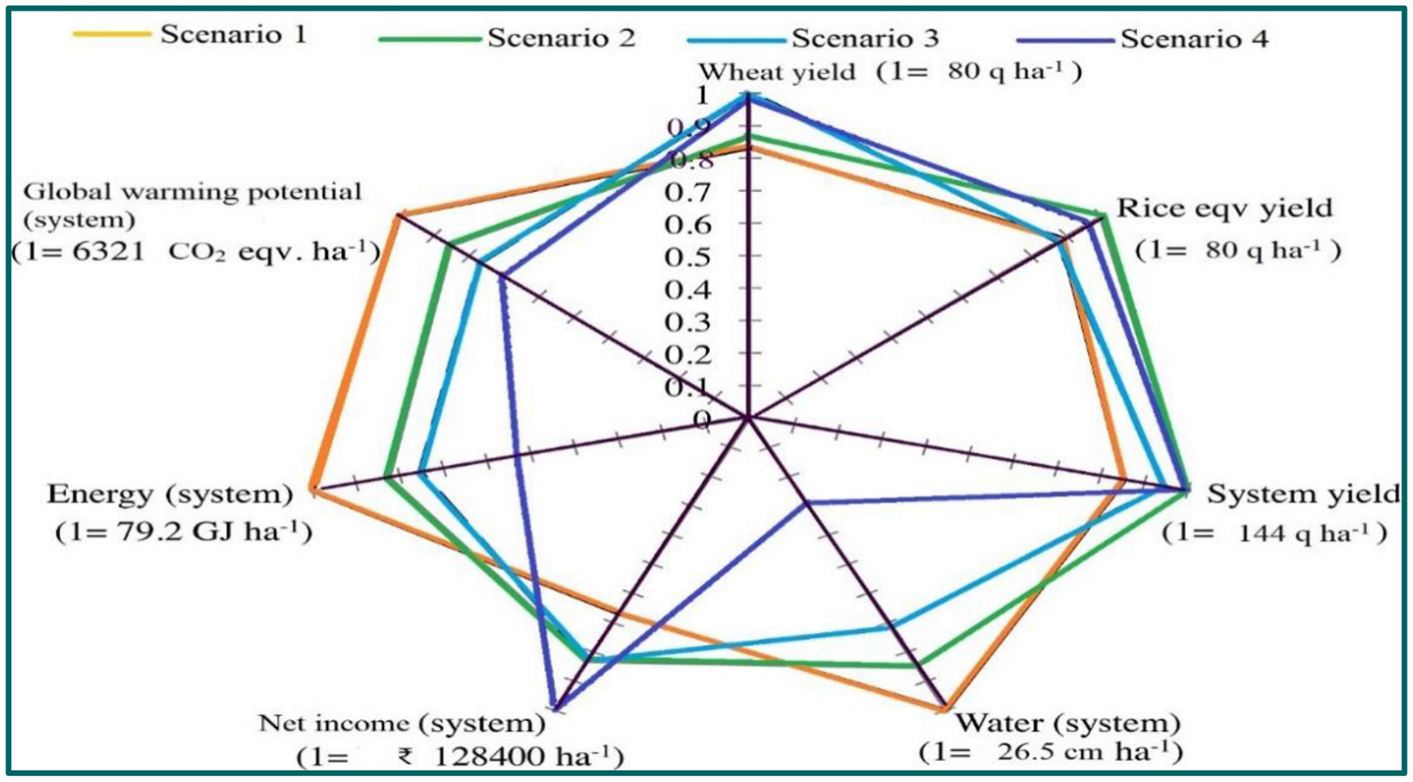
Several indicators of assessing the long-term performance of diverse scenarios in IGP at Karnal, India. The reduced–till rice–wheat–mung bean system (scenario 2) produced considerably greater system yield (17%), net return (24%) with 17, 15, and 15% fewer energy inputs, irrigation water, and global warming potential (GWP) over conventional rice–wheat–fallow system (scenario 1). By shifting from scenario 2 to CA-based rice–wheat–mung bean system (scenario 3) there was further saving of 9, 15, and 10% energy inputs, irrigation water, and GWP with similar profitability but yield reduction of 8 q ha^−1^. The shifting from scenario 3 to scenario 4 (CA-based maize–wheat–mung bean system) gave 21% more net income than scenarios 2, 3, and 5% more system yield than scenario 3 with 29–35% fewer energy inputs, 59–66% less irrigation water, and 8–18% less GWP than scenario 2 and 3 (Modified; Kumar et al., 2018). Variable means are normalized on a 0–1 scale, where 1 represents the greatest absolute value of that variable. The highest absolute value is also shown for each parameter.

The sustainability and production economics of a cereal-based system can be significantly enhanced by the horizontal expansion of areas by bringing the fallow periods under cultivation of short-season pulse crops in a rainfed agroecosystem. In addition, to improve the grain and protein yield of the subsequent non-pulse crop, diversified crop rotation also ensures the sustainability of land use and production system. Three types of the fallow period are there in rainfed agroecosystems, *viz*., summer fallow or pre-*Kharif* fallow (April–June), rice fallow (November–February), and fallow period in drylands (*Kharif* fallow in the absence of adequate rainfall or *rabi* fallow due to lack of residual moisture). After harvesting the *rabi* crops and planting *Kharif* crops, there is a summer fallow of ~80–90 days. Summer fallowing leaves land unprotected and bare, deprived of any crop planted for one growing season, generating a chance to lower production in a rainfed agroecosystem (Gan et al., 2015). In addition, summer fallowing has heavy soil and environmental consequences. Therefore, an alternative production system needs to be identified that can overcome the negative impacts of summer fallowing and redeem positive benefits apart from advancing system productivity (Ghosh et al., 2019). Shifting this summer fallow toward pulses in the cereal-based system is a win–win situation in terms of system productivity, net profitability, water productivity, efficient energy utilization, and reduced global warming potential (GWP) (Figure 7) (Kumar et al., 2018).

This short period could be diversified and effectively utilized by taking short-duration pulse crops such as mung bean, cowpea, and urd bean, under the rainfall, irrigation facilities, and production cost (Gan et al., 2015). In this context, Ghosh et al. (2019) noted 10–14 and 5–11% improvement in grain yield of rice and wheat, along with 70 and 79% higher system productivity in lowland rice–wheat and maize–wheat systems, respectively, with the inclusion of pulses (mung bean) in the summer fallow period (April–May) at Kanpur, India. The replacement of wheat with chickpea in the lowland rice–wheat system also improved rice grain yield by 5–8%. Long-term experiments are an effective tool for identifying system productivity, compatibility of component crops, stability, and economic efficiency. Considering this, after analyzing for 30 years, St. Luce et al. (2020) reported that the wheat–canola–wheat–field pea system gained 14–38% higher economic yield concerning continuous wheat and summer fallow–wheat–wheat system in semi-arid regions of Saskatchewan, Canada. This shows that diversifying existing summer fallow and monoculture with pulses has better fallow utilization, better soil health, improved protein-rich yield of the subsequent crop, and bonus yield from pulses that provide nutritious and health-conscious food and economic advantages (St. Luce et al., 2020; Wang et al., 2020). Although, for promoting pulses in summer fallow, adequate availability of quality seed, reducing production costs, managing labor costs, and optimizing production techniques must be accomplished. Farmers accept and adopt any technology only when it is economically flexible. Furthermore, it is also vital to select the pulse crop to optimize resource utilization by considering the suitability of the agroecosystem. Although, changing the existing agroecosystem may face other challenges in addition to the inadequacy of irrigation facilities which is the major challenge obstructing the cultivation of summer pulses in cereal-based rotation in the rainfed ecosystem. Growing mung bean in summer fallow demands frequent irrigation due to high evaporative demands that are not sustainable, resulting in a rapid decline in groundwater. Although, black beans could be a better option during March–May for SI, specifically in rice–maize, rice–potato, etc., in eastern India (Hazra and Bohra, 2020). Therefore, this new system needs an assessment of resource use efficiency of water, N, and ecological footprint in each unit of land and production system. Still, the widespread adoption of summer pulses, instead of leaving fallow, will require finding more reasons for the non-adoption of this system and focusing on solutions through adaptive research in different agro-ecological regions.

India alone has ~11.695 Mha fallow area out of 22.2 Mha of entire fallow regions of south Asia after harvesting rice. Of this, ~82% falls in the Eastern States, including West Bengal, Odisha, Jharkhand, Assam, Bihar, Chhattisgarh, and Eastern Uttar Pradesh, and the remaining lies in Peninsular India, *viz*., Karnataka, Tamil Nadu, and Andhra Pradesh (IIPR, 2016). These areas are from where rice is taken in the monsoon season (June to October), while the period between November and February remains uncultivated. This is due to the lack of irrigation and good rainfall, low residual moisture, excessive moisture in December, soil compaction and cracking in vertisols, growing long-duration rice varieties, lack of appropriate pulse varieties, and the problem of stray animals. Short-duration and high adaptive capacity with fewer inputs under climate change fit pulses best in these fallows to increase the overall system productivity (Table 8).

**Table 8:** Potential pulse crops and potential varieties for rice fallows in different states (NAAS, 2016; Singh et al., 2017).

**Crop**	**States**	**Suitable varieties**
Lentil	Assam, West Bengal, Bihar, Odisha, Eastern Uttar Pradesh, Chhattisgarh, and Jharkhand	Narandra Masoor 1, WBL 58 KLS 218, HUL 57
Pea	Jharkhand, Chhattisgarh, Eastern Uttar Pradesh, and Northern Madhya Pradesh	IPFD 10–12, IPFD 11–15, KPMR 400, Prakash, DMR 57 and 11, Malviya Matar 15, Rachana
Chickpea	Chhattisgarh, Bihar, Uttar Pradesh, Jharkhand, West Bengal, and Madhya Pradesh	Pusa 372, Pusa 547, JG 14, JG 16, Rajas, Pant G 186, GCP 105
Mung bean	Odisha, Andhra Pradesh, Tamil Nadu, and Karnataka	IPM 2–14, Virat, Shikha, HUM 16, TU 40, VBG 04-008, LBG 787, TM 2000-2
Urd bean	Coastal Andhra Pradesh, Tamil Nadu, Karnataka and Odisha	LBG 402, LBG 752, LBG 709, Pant U 31, IPU 2-43
Grass pea (Lathyrus)	Tal area of Bihar, Chhattisgarh, and West Bengal	Prateek, Ratan, Mahateora
Lablab bean	Andhra Pradesh, Tamil Nadu, Karnataka, Jharkhand and Bihar	Arka Vijay, Pusa Sem 2 and 3, CO 12, 13, and 14, KDB 405

Therefore, diversifying existing central cereal-based systems with the inclusion of pulses during the fallow period could prove a trump card for sustainable yield, food security, and restoration of soil health in a rainfed agroecosystem (Sravan and Murthy, 2018). Taking another crop in rainfed rice fallow has a lot of challenges, including unfavorable soil properties, due to puddling, rapid residual soil moisture depletion, unavailability of appropriate cultivars, socioeconomic status of farmers, and lack of awareness among them (Kumar et al., 2017d; Hazra and Bohra, 2020). Pulses help eradicate rice production's existing constraints, such as depleting natural resources, yield stagnation, and other soil and environmental issues, which may facilitate economic development and alleviate hunger and poverty. The traditional relay cropping can be followed in eastern India, where long-duration rice cultivars are grown, wherein pulse seeds are broadcasted in standing rice 10–12 days before rice harvesting, permitting efficient utilization of the residual soil moisture. The rotation of pulse crops in rice-fallow needs locally adapted short-duration rice cultivars, water-efficient and early maturing pulse cultivars, and farm mechanization (zero tillage machinery). The rice fallow areas are not much explored; therefore, breeding and identifying new varieties suited to the soil and agroecological conditions, improved management practices, and technological intervention are primarily essential. For this, there is a strong need for a robust database and associated human, organizational, and governmental support in the agronomic management of pulses, which are highly exposed to climate change as pulses are crops of a rainfed agroecosystem (Lal, 2017).

The formulation and implementation of an appropriate scheme have a great role in exploring these fallow lands for cultivating pulses using improved management technologies. Recently, the Indian government has taken outstanding initiatives to screen and identify pulse crops and their cultivars, agronomic management, and other promotional technologies. To promote pulse cultivation in these fallow areas, recently in 2016–2017, a scheme Targeting Rice Fallow Areas (TRFA) was implemented under Rashtriya Krishi Vikas Yojana (RKVY) in eastern India by the Department of Agriculture Cooperation and Farmers Welfare (DAC&FW) targeting 4.5 M ha rice fallow to bring under winter pulses in the next 3 years by releasing a fund of ₹ 75 crores (US$ ~10.3 million) (NAAS, 2016). In 2016–2017, the TRFA was initiated in 15 districts of 6 states (West Bengal, Odisha, Jharkhand, Chhattisgarh, Bihar, and Assam) to cover 19.14 lakh ha areas. In 2017–2018, the government extended the areas of the scheme to 40 districts and 4,000 villages to cover 15 lakh ha with the support of Minikit distribution, cluster demonstration, and farmers' training (MoA&FW, 2018). Most rice fallows were covered with lentils, peas, mung beans, chickpeas, urd beans, pigeon peas, and lathyrus. The government is further planning to extend the TRFA program in the rice fallows of southern, northeastern, and Himalayan states by the year 2022 with new norms of assistance (MoA&FW, 2019). Several institutional and organizational projects are ongoing in different regions of the country to strengthen pulse cultivation in fallow soils. The government is also promoting areas under pulse cultivation to enhance their production, specifically lentils, chickpeas, and lathyrus in rice fallow through another plan, National Food Security Mission-Pulses (NFSM-Pulses). The NFSM-funded projects-CG institutes are International Crops Research Institute for the Semi-Arid Tropics (ICRISAT)—Enhancing chickpea production in rainfed rice fallow land of Chhattisgarh and Madhya Pradesh (2008), International Center for Agricultural Research in the Dry Areas (ICARDA)—Expanding lentil production in Eastern and Northeastern states under rice-based production system in India (2010), and ICARDA—Enhancing grass pea production in Eastern and Northeastern states under the rice-based production system (2010). The farmers are encouraged to grow pulses under the NFSM through cluster demonstration, quality seed distribution, arranging for improved machinery/tools/technologies, water application tools, and other facilities. To support this scheme, other governmental plans like Mahatma Gandhi National Rural Employment Guarantee Act (MGNREGA), RKVY, and Pradhan Mantri Krishi Sinchayee Yojana (PMKSY) are also contributing by developing rainwater harvesting structures like farm ponds, which can be used further as supplemental irrigation to enhance the survival rates and productivity of pulses in rice fallow. In addition, other research and development activities undertaken for promoting pulse cultivation in rice fallow are the All India Coordinated Pulse Improvement Project (AICRP) (1967) on Mung bean, Urd bean, Lentil, Lathyrus, Rajmash and Pea (MULLaRP) (1995), mitigating abiotic stresses and enhancing resource-use efficiency in pulses in rice fallows (2010), and brainstorming meeting on rice fallows (2013). Pulses are climate smart as they simultaneously adapt to climate change and contribute toward mitigating its effects. Therefore, introducing them to farming systems can be vital to increasing resilience to climate change through diversification of the income source, increased stability to climate extremes, and increased productivity.

## 6. Adaptation potential of pulses to climate change

Climate change, food production, and food security are interconnected; therefore, it is impossible to evaluate them independently. Climate change gives rise to several abiotic stresses, including drought, cold, heat waves, flood, submergence, high temperature, and increased attacks of insects, pests, and diseases (Kumar et al., 2019c; Sheoran et al., 2022b). This climate change is putting an extra burden on already water-stressed systems and will intensify the competition for water with other sectors. Heat stress and water scarcity are probably the most acute stresses faced by the agricultural production system (Pereira, 2016), which will be aggravated more with the predicted increase in temperature by 2–4°C over the next 100 years in a rainfed agroecosystem. Pulses have an inherent capacity to adapt and perform well under climate change because of their hardy nature to fight abiotic stresses (Table 9).

**Table 9:** Adaptive traits of pulse crops to climate extremes.

**Pulse crop**	**Niche**	**Adaptive traits**	**References**
Chickpea	Semiarid tropics and dry areas	Heat and water-stress tolerant; resistance to pod borer, Fusarium wilt, A*schochyta* blight, *Botrytis* gray mold, and root rot	Whish et al. (2007)
Pigeon pea	Tropical climate, semiarid and less humid regions	Short stature; resistance to Fusarium wilt, *Phytophthora* stem blight, sterility mosaic virus, pod borer, and pod fly	Kumar et al. (2019a)
Mung bean	Arid and semiarid regions, warm-season	Very short duration, photo-and thermal insensitive; heat and water-stress tolerant; favors stressed conditions; resistance to powdery mildew and mung bean yellow mosaic virus	Tickoo et al. (2006), Gupta et al. (2019)
Moth bean	Arid regions, warm climate	Early and synchronous maturity, erect plant habit; tolerant to heat and water stress; grown in low soil fertility; tolerant to yellow mosaic virus	Tiwari et al. (2018); Gupta et al. (2019)
Urd bean	Semiarid regions, hot humid season	Very short duration, photo-and thermal insensitive; tolerant to excessive moisture stress; resistant to powdery mildew and mung bean yellow mosaic virus	Gupta et al. (2019)
Cowpea	Wider adaptation, arid and semiarid regions	Early maturity, fast initial growth, and better source/relationship	Kumar and Dixit (2003); Hall (2012)
Pea	Semiarid climate, cool areas	Dwarfness, tendril, leaflessness, and earliness; tolerant to terminal heat and water stress; resistance to powdery mildew and rust	Kumar and Dixit (2003)
Lentil	Semiarid tropics and dry areas	Heat and water-stress tolerant; resistance wilt, A*schochyta* blight, Stemphylium blight, rust, and black aphid	Sarker and Erskine (2002)
Lathyrus	Indian and Mediterranean region	Tolerant to both excessive water and drought conditions	Kumar and Dixit (2003); Kumar et al. (2019a)
Horse gram	Arid and semiarid regions	Early maturity, green foliage up to maturity; thermo-insensitive, tolerant to water stress and soil acidity	Kumar and Dixit (2003); Kumar et al. (2019a)
Common bean	Tropical climate	Short height, early maturity; tolerant to heat, water stress and cold; resistant to common bacterial blight	Kumar and Dixit (2003); Kumar et al. (2019a)
Lima bean	Climate of Mexico, Argentina, and Georgia	Bushy compact plant, climbing type; well suited under limited water and marginal soils	Kumar and Dixit (2003); Kumar et al. (2019a)
Rice bean	Dry regions of arid and semiarid climates	Determinate growth habits, early maturity; tolerant to water stress and soil acidity	Isemura et al. (2010); Kumar et al. (2019a)
Tepary bean	Tropical climate, dry season	Short duration, deep-rooted crop; resistance to water stress and common bacterial blight	Kumar and Dixit (2003); Kumar et al. (2019a)
Adzuki bean	Temperate and sub-tropical climate	Tolerant to water stress; resistance to bacterial blight	Kumar and Dixit (2003); Kumar et al. (2019a)
Hyacinth bean	Sub-humid and semiarid regions	Early maturity; tolerant to water stress and soil salinity	Kumar and Dixit (2003); Kumar et al. (2019a)

Pulses can withstand harsh conditions and survive well under water scarcity since they require fewer inputs than input-intensive cereals. Therefore, they play an essential role in climate change adaptation as water becomes scarce and the temperature rises. On a global scale, many neglected pulse species can be used for specific niches because of their adaptation to marginal environmental conditions. Therefore, pulses will be a game-changer in the impending climate change era, owing to their capacity to supply nutritious food under punitive soil and ecological requirements. Globally, pulses, beans, and cowpea demands are expected to increase by 155% from 2015 to 2050 (Kissinger, 2016). Despite India being the world's largest producer of pulses in the world, the country will need to increase pulse production by 32 and 39 million tons by 2030 and 2050 to fulfill the fast-growing population's domestic needs (Dixit et al., 2015). Therefore, to attain the targeted food production under the growing stresses of climate change, pulses are the first choice of farmers and scientists for enhancing climate change resilience as they adapt better to changes in climatic stimuli (FAO, 2016a).

Pulses differ in morphophysiological traits and have wider adaptability than cereals; naturally, they are drought-tolerant crops and can sustain and produce an economic yield in the rainfed agroecosystem. Pigeon pea is one of the most drought-tolerant pulse crops that can also be produced under drought spells. The ability of pigeon peas to perform even under drought conditions is attributed to a higher osmotic adjustment in leaves (0.1–1.6 MPa; mega pascal) (Basu et al., 2018) and a deeper root system. Osmotic adjustments help the pigeon pea plant to moderate stomatal conductance and photosynthetic functions, even at low water potential, diminish floral dropping, delay leaf senescence, and improve the rooting system and water extraction from deeper soil strata (Basu et al., 2018). Some of the identified pigeon pea varieties having higher osmatic adjustments and adaptive capacity to drought conditions are AL 201, AL 1855, TGT 501, BSMR 853, BDN 708, BDN 2008-12, ICP 13673, ICP 84031, VKS11/24-1, VKS11/24-2, GRG 815, GRG 2009-1, JKM 7, MAL 13, RVK 275, Bahar, and Bennur Local (Basu et al., 2018). Naturally, lentils can grow well under fewer water conditions, and even supplying irrigation/rainfall before the reproductive stage of the crop causes significant yield losses. It can withstand water-stressed conditions through drought avoidance and tolerance mechanisms (Gupta et al., 2019). Its drought avoidance mechanisms involve the availability of short-duration cultivars and associated early flowering, faster early crop growth, extensive rooting network, and higher yield potential. In addition, various morphological features, such as leaf surface, length, orientation, stomatal behaviors, and canopy structure, pay to drought avoidance mechanisms for climate change adaptation (Biju et al., 2017). Early maturity of lentil cultivars helps to mature and set seeds before the commencement of terminal heat, water stress, and resultant enforced maturity that causes yield loss. Recently, efforts have been made by ICARDA to develop short-duration lentil cultivars to escape from terminal heat stress, such as BARI M 5, BARI M 4, BARI M 6, Idleb 3, Bakaria, and Precoz, without any yield reduction (Basu et al., 2018). While the drought tolerance mechanisms show dense leaf pubescence, a high osmotic adjustment of the leaf (0.6 MPa), controlled stomata closure, and increased response of antioxidants (Gupta et al., 2019).

Furthermore, the wild landraces of lentils are more resilient to climate change and can be included in the breeding program of lentils to enhance their adaptive capacity to climate change. In chickpeas, a higher osmotic adjustment (0–1.6 MPa) and the production of glandular hairs are important adaptive traits for delaying the effects of drought, either by reducing stomatal density and/or transpiration or by increasing leaf reflectance that helps in more interception of non-photosynthetic radiations (Basu et al., 2018). Even these winter pulses (e.g., chickpeas and lentils) require physiological stress (i.e., drought) to terminate flowering and induce seed set. Summer pulses, such as mung bean and moth bean, do not perform well under continuous water supply and reduced temperature. They require stressed conditions (i.e., water stress and temperature stress) for their outstanding performance, which will arise because of climate change. Hence, mung bean and moth bean, being the warm-season crops, are expected to be less affected by high temperatures resulting from climate change. In urd bean, the presence of a waxy cuticle above the epidermis coupled with dense hairs of the pod wall reduces moisture loss. Thus, the pod continues photosynthetic activities under stressed conditions. The anatomical difference in urd bean imparts drought tolerance to climatic extremes like high temperature and drought.

Pulses provide opportunities to sustainably intensify the existing agroecosystem and help to moderate the risk associated with unpredictable weather and other market issues. Food legumes, such as pigeon peas and mung bean, are a better option for inclusion in the agroforestry system for sustaining soil health, soil C sequestration, food security, and the economics of farms by diversifying sources of income (Roy et al., 2021). This system can stand more strongly against climatic extremes because of the hardier nature of pulses, along with benefitting the soil system (FAO, 2016b). Including pulses in mixed and intercropping systems conserves soil moisture and improves water availability, and thus is an excellent example of high-priority adaptation strategies to conditions of rainfall variability in a rainfed agroecosystem. Similarly, including at least one pulse crop in a cereal-based cropping system, either as an intercrop, sequential crop, or relay crop, enhances the adapting capacity of the system to climactic adversities. In addition to their excellent adaptation capacity, these crops also greatly aid in reducing greenhouse gas (GHGs) emissions, owing to their superb mitigation potential.

## 7. Climate change mitigation potential of pulses

Pulses in the agroecosystem contribute to climate change mitigation through GHGs emission reduction and SOC sequestration (Lal, 2017). Production and application of synthetic fertilizers in the agricultural production system are the major contributors to GHGs emissions, whose share in emissions is increasing at the rate of 4% per year from 1961 to 2010 (Smith et al., 2014). In field crops, N fertilization contributes 36–52% of total emissions, which can be significantly reduced by diversifying the existing agroecosystem with pulses to enhance fertilizer use efficiency. Diversification cuts the C footprint of the system by 32–315% compared to continuous monoculture (Liu et al., 2016). Pulses not only have a lower need for nitrogenous fertilizers, but they also cater to the N needs of the subsequent non-legume crop in rotation, and thus, the emission of GHGs is reduced. In addition, less tillage requirement and associated fuel-saving, low irrigation requirement and related water and fuel/energy saving by pumping, and reduced herbicide application due to rapid initial ground cover through faster foliage growth (e.g., cowpea, mung bean) also reduce the emission of GHGs. To confirm the assumption, in western Canada, MacWilliam et al. (2018) tested a pulse-based cropping system under two treatments, *that is*, thoroughly recommended application of synthetic N and reduced dose of synthetic N fertilization to subsequent cereal or oilseed. They observed that the emission of GHGs from cereal and oilseed crops grown after lentil and dry peas was much higher (286–598; 888–987 kg CO_2_ eq. t^−1^) with complete synthetic N application than that from reduced N application (116–598; 311–978 kg CO_2_ eq. t^−1^) without compromising yield. This suggests that emission can be significantly reduced by growing pulses in rotation as a preceding crop as these crops offset the N fertilizer requirements of following non-legume crops and thus an overall reduction in synthetic N application. In the silt loam of Saskatchewan, Canada, Gan et al. (2011b) assessed the C footprint of durum wheat by diversifying the cropping system with pulses (e.g., chickpea, lentil, and dry peas). They found 37% reduced emissions (162 kg CO_2_ eq. ha^−1^) in a pulse–durum wheat system compared to cereal/oilseed–durum systems (251 kg CO_2_ eq. ha^−1^) due to reduced fertilizer application. Therefore, the C footprint of the pulse–durum wheat system was 28% (0.30 CO_2_ eq. kg^−1^ of grain) lower than cereal–durum rotation (0.42 CO_2_ eq. kg^−1^ of grain). At Swift Current, Saskatchewan, Campbell et al. (2000), based on a long-term study of 17 years, reached the result that CO_2_ eq. emissions from N_2_O (nitrous oxide) emissions and energy inputs were 180 and 170 kg ha^−1^ lower for wheat–lentil and wheat–field pea rotation, respectively, over the spring wheat monoculture. By making the same assumptions, Zentner et al. (2002) found a reduction of CO_2_ eq. emissions by 160 or 40 kg ha^−1^ net SOC reduction upon inclusion of chickpea over the one that did not. The result of another experiment (Gan et al., 2011b) suggested that durum wheat grown after pulse crops (e.g., chickpea, lent, and dry peas) released total GHGs of 673 kg CO_2_ eq. that was 20% less when durum wheat was grown after cereal crop. The C footprint of durum wheat preceded by pulses was 0.20–0.30 (averagely 0.25) (kg CO_2_ eq. kg^−1^ of grain) were 32% lower than durum wheat grown after the cereal crop. Pulses in rotation also reduced the C footprint (kg CO_2_ kg^−1^ of grain) of canola (0.80), mustard (0.59), spring wheat (0.46), and chickpea, lentils, and dry peas (0.20–0.30) (Gan et al., 2011a).

The N_2_O emission under pulse cultivation may be more than that released under the cereal field, which must be adequately managed. Jain et al. (2016) quantified the GHGs emission under pulses, oilseeds, millets, and cereals in northwestern India. The analyzed data showed that the total amount of N_2_O emissions in pulses, oilseeds, millets, and cereals was 67, 55, 43, and 40% of applied N, respectively. Sah and Devakumar (2018) analyzed the data for five decades (1960–2010) and found the maximum emission of 23.75 Tg C eq. ha^−1^ was from rice fields while it was the lowest from pulses, *for example*, pigeon pea with a value of 2.98 Tg C eq. ha^−1^. Similarly, Jain et al. (2016) measured the lowest C equivalent emission in pigeon pea (833 kg C ha^−1^), while it was the highest in wheat (1,042 kg C ha^−1^). However, the GWP for pigeon peas and wheat was 3.05 and 3.97 tons CO_2_ eq. ha^−1^, respectively, but the GWP per unit of seed yield was the highest in pulses and the lowest in cereals might be due to a higher emission of N_2_O in pulses.

Pulses promote soil C sequestration by improving soil organic stock contributed by fallen leaves, straw, roots, rhizodeposition, *etc*., and by improving microbial biomass carbon (MBC) (Kumar et al., 2018). A significant fall of 3.0 and 1.67 Mg ha^−1^ leaves (dry weight) was found in pigeon pea crops sown during rainy and postrainy seasons, respectively. In addition, chickpeas and lentils also have a high leaf fall during senescence. The way of the addition of leaf C to the soil can significantly contribute to the soil C stock. In IGPs of India, Venkatesh et al. (2013) observed that the diversification of the existing maize–wheat system toward a more remunerative pulse-based system, *that is*, maize–wheat–mung bean or pigeon pea–wheat significantly improved the SMBC by 10–15%. In fine sandy soils of the south-central portion of Washington, Wang et al. (2012) found that the growing of faba bean added a maximum amount of soil C (597 g C m^−2^) into the soil compared to other winter crops, purple vetch (378 g C m^−2^), triticale (369 g C m^−2^), ryegrass (342 g C m^−2^), mustard (247 g C m^−2^), and white bean (149) g C m^−2^. In IGPs, Venkatesh et al. (2013) revealed that pulses (e.g., mung bean and pigeon pea) in rotation had nearly 10% more organic C level in soil and C management index than those obtained without pulses, *that is*, maize–wheat system. In IGPs, the inclusion of pulses in the traditional puddled rice–wheat system improved the soil C flux and MBC by 6 and 85% as regards the conventional practice of a continuous rice–wheat system (Ghosh et al., 2012). In Asia, also the growing of mung beans in the summer fallow of the existing rice–wheat system produced an additional 4.5 Mg ha^−1^ dry matter that directly contributed to the soil C pool (Yaqub et al., 2010). In the black soils of semi-arid tropical India, Chaudhury et al. (2016) observed that monocropping of cotton had no C addition to the soil. Still, the organic and inorganic C increased by 0.75 and 1.12% upon rotation with pigeon peas. Pulses have a positive impact on the emission reduction of GHGs, but still, it is small. The improved N availability and associated soil quality under pulses in rotation improve the soil's organic status, which helps to reduce GHGs emissions. Although, the buildup of the C pool in soil dramatically depends upon the pattern of C translocation among different species. For instance, pasture crops translocate 30–50% of biomass belowground, equivalent to around 2.2 Mg C ha^−1^; in cereals, this figure is 20–30%, corresponding to ~1.5 Mg C ha^−1^ (Kuzyakov and Domanski, 2000). The C:N (CN ratio) of pulse crop root, shoot, and seed are also considered crucial determinants for enhancing soil C stock. Usually, the CN ratio of pulses stover (e.g., lentil, chickpea, and peas) is ~17 compared to wheat (~32) and oilseeds (e.g., mustard, canola, and linseed) (~41). Likewise, root C mass in the top 20 cm significantly differed with crop species as 41% for chickpeas, 55% for lentils, peas, linseed, *etc*., while in mustard, wheat, and canola, it was 66% (Gan et al., 2011b). In search of water and nutrients, chickpea roots got more profound in the soil profile, which after harvesting, leave root residues at a greater depth and thus are less prone to microbial decomposition. Ghosh et al. (2012) found that in the rice—wheat system of IGPs, taking chickpeas, instead of wheat, totally or as a minimum in alternate years added more organic C to the soil over the rice–wheat system. So, the extensive root system of pulses is the critical factor in conserving available natural resources, including water, along with an increased nutrient uptake and associated energy conservation.

## 8. Pulses for enhancing resource use efficiency

Soil, water, energy, nutrients, air, biodiversity, forest, watershed, and vegetation are significant gifts of nature whose conservation is a must to sustain crop production. The degradation of these resources is an important environmental issue that drastically affects the biological system and sustainability of agroecosystems throughout the globe (Kumar et al., 2018). The misuse of soil and water resources makes the production system sick, leading to a reduced response of applied inputs such as fertilizer, irrigation, agrochemicals, tillage, *etc*. At the same time, climate change threatens crop production, causing significant challenges to food security and efficient resource management (Bajiya et al., 2017; Lakhran et al., 2017). Fulfilling the requirements of growing food demand and efficient resource management will not be accessible without significant changes to the existing production systems for long-term ecological sustainability. Pulses have become the brand ambassador of resource-efficient agriculture in rainfed agroecosystems, owing to their limited demands of water, energy, and nutrients along with C-efficient and ability to grow well in poor and marginal soils. These crops also get a high net return to the farmers with the reduced cost of cultivation and higher marketing price.

Diversifying the existing agroecosystem with pulses is more productive and profitable and bridges the gap between food and nutrition, while being resilient to extreme climatic events. Pulses are water-efficient crops as they consume less water and have the capacity to grow on residual soil moisture, such as chickpeas and lentils, in northern and central India, and mung bean, urd bean, cowpea, chickpea, lathyrus, and lentil in rice fallows of southern, eastern, and northeastern India during November to February. At the same time, winter pulses (i.e., chickpeas and lentils) can be grown with only one irrigation in IGPs, whereas at the same time, the wheat crop requires 5–6 irrigations (Kumar and Yadav, 2018). Pulses have lesser water demand and higher WUE than cereal, oilseeds, and other commercial crops because of their morphological and physiological characteristics, as described in section 7 and Table 9. The water requirement for rice is 100–200, 30–40 cm for wheat, and 150–250 cm for sugarcane, while pulses need only 15–25 cm of water (Kumar and Yadav, 2018). Therefore, with the same quantity of water, ~4.8 times more areas can be occupied under pulses than rice. Cereals consume ~60% of global water, while pulses consume only 4% of global water (Kumar et al., 2018). Pulses on average require only 2,500 gallons of water to produce one ton of seeds, while an equal amount of eggs, chicken, pork, and beef is being produced by ~3,200, 4,500, 5,900, and 20,700 gallons of water, declaring them very water-efficient crops. In this line, the production of one kilogram of *dal* (pulse splits) requires ~1,250 liters of water, while the output of the same quantity of chicken, mutton, and beef requires 4,325, 5,520, and 13,000 liters of water (FAO, 2016a), hence, pulses are climate-smart crops with few water requirements. The more profound and extensive network of roots enables them to extract moisture from deeper soil sections, strengthening their survival ability in the rainfed system. In silt loam soils of Swift Current and Stewart Valley of Saskatchewan, Canada, Gan et al. (2009) found that the average WUE of pulse crops (*i.e.*, chickpea, lentil, and dry peas) was 4.08 kg ha^−1^ mm^−1^, significantly more than oilseeds (3.64 kg ha^−1^ mm^−1^) and spring wheat (5.5–7.0 kg ha^−1^ mm^−1^). They also critically observed that the pulses removed most of the water from the top 0.6 m of soil and left behind more than wheat and oilseeds. This unused and conserved soil moisture could be used better by a deep-rooted crop to be raised after pulses which may improve the WUE of the entire cropping system in rainfed environments. In another study at Swift Current, Saskatchewan, Canada, Miller et al. (2001) also observed the improved WUE in pulse crop (dry peas) (9.1 kg ha^−1^ mm^−1^) concerning the wheat (6.4 kg ha^−1^ mm^−1^) under fallow and stubbled-crop conditions. The WUE of dry peas varied from 59 to 186% (averagely 132%) compared to wheat crops due to the higher productivity of dry peas and reduced water withdrawal below 0.6 m soil section (Wang et al., 2012). The study by Wang et al. (2012) suggested that among pulses, dry peas had the greatest WUE with an average value of 8.3 kg ha^−1^ mm^−1^ while the lowest was in chickpeas (5.62 kg ha^−1^ mm^−1^). Similarly, in Mediterranean types' conditions of South-eastern Australia, Siddique et al. (2001) found that the maximum WUE for seed yield in peas was up to 16 kg ha^−1^ mm^−1^, while the WUE for dry matter production in faba bean and narbon bean was maximum with a value of 30 kg ha^−1^ mm^−1^.

**Figure 8:**
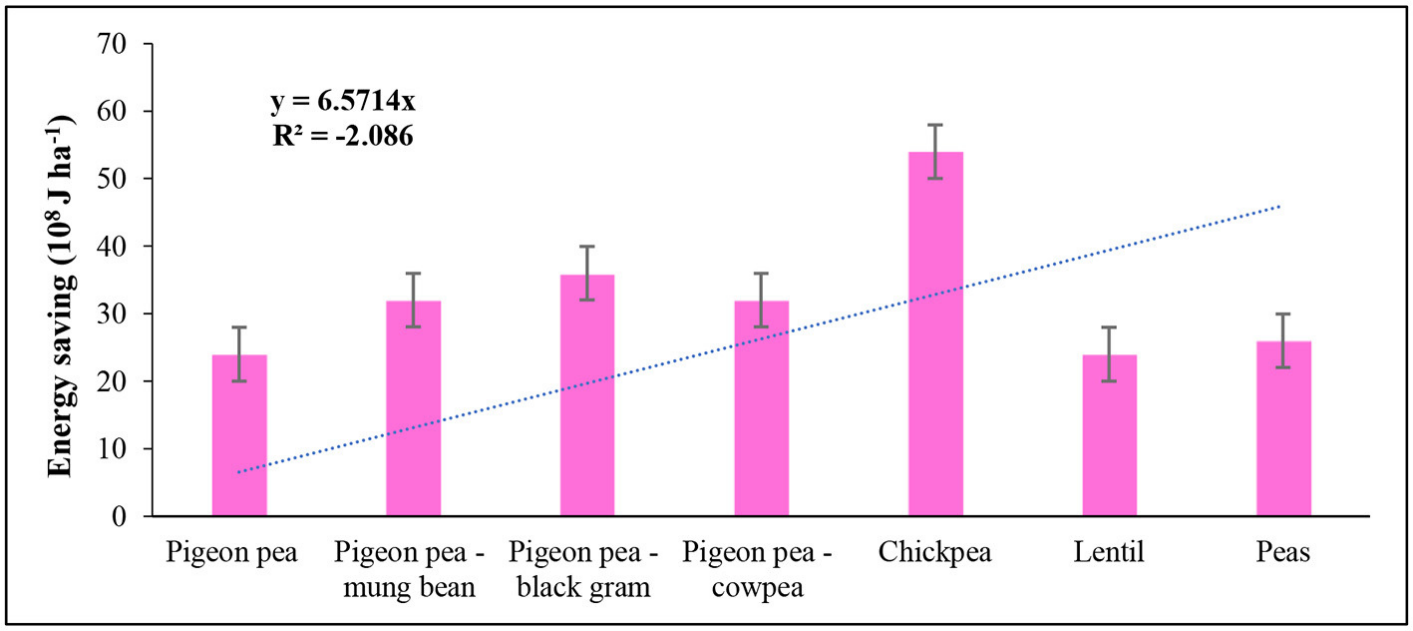
Quantum of energy saved by pulse crops through *N* economy (*y* = 6.5714*x, R*^2^ = *0.7475*). Sole cropping of chickpea saved maximum energy (54 × 10^8^ J ha^−1^) by saving 68 kg N ha^−1^ followed by pigeon pea–urd bean rotation (36 × 10^8^ J ha^−1^), while the lowest energy saving was observed from both lentil and pigeon pea (24 × 10^8^ J ha^−1^ each) having N economy of 30 kg N ha^−1^ (*Data source:* Ahlawat and Srivastava, 1997; Kumar and Yadav, 2018).

Integration of pulses into cropping systems enables the fixation of free atmospheric N into a plant-available form for their own use and then hands over the leftover N to the companion crop. This ensures a better utilization of nutrients under intercropping systems through complementary interaction and niche facilitation in the rooting zones of companion crops, reducing competition among component crops for nutrients, and thus enhancing efficiencies of nutrient use (Gitari et al., 2018; Punia et al., 2020). Likewise, the roots of pulse crops exude particular types of chemical substances that can solubilize native P in soil by replacing the P from the exchange site, thus increasing its availability in soil solution, which the intercropping system can efficiently utilize. The higher N use efficiency (NUE) of pulse crops is associated with the gathering and accumulation of N in their plant system rather than the release in the atmospheric system (Hocking and Reynolds, 2012; Kumar et al., 2022b). The improved NUE reduces their application through fertilizers and minimizes groundwater pollution and the circulation of chemical ions in the food system, ensuring healthy food production. Gan et al. (2015) in Saskatchewan, Canada, in a 3-year pulse-based cropping cycle, found that diversifying summer fallow with pulse crops enhanced the fertilizer-N use efficiency (NUE_f_) by 33% over the summer fallow in rainfed dry areas. The pulse cropping system improved the NUE_f_ by 36.6 and 62.6% for seed and protein yield, respectively, over the fallow period. Similarly, compared to the cereal monoculture, the improvement in NUE_f_ for seed and protein yield was 99 and 186.6% in the pulse system. Deep-rooted pulses such as lablabs have the capacity for a better exploration of water and nutrients from deeper soil horizons, so competition for these resources in the surface profile is reduced, and shallow-rooted crops such as potatoes get the benefit. Using the concept, in Nairobi, Kenya, Gitari et al. (2018) reported a significant improvement in N and phosphorus use efficiency (NUE and PUE) in pulse + potato intercropping over sole potato planting. The NUE of potato + lablab, potato + bean, and potato + peas was 30, 19, and 9% greater than that of the sole potato, respectively, while in the case of PUE, this improvement was 6, 14, and 21% compared to that of potato monoculture. In the semi-arid regions of Saskatchewan, Canada, St. Luce et al. (2020), in a long-term experiment of 30 years, concluded that the wheat–canola–wheat–field pea system had greater NUE_f_ (26.4 g kg^−1^) and NUE (4.1 g kg^−1^), compared to those attained from green manure–wheat–wheat system (18.1 and 2.7 g kg^−1^, respectively). Fertilizer is the most significant proportion of total energy inputs whose production, packaging, transportation, and application consume a lot of energy. A third of the total energy input to crop production goes to the production of fertilizers, one-third to mechanization, and one-third to other inputs, including electricity, labor, pesticides, and transportation. According to a study, diesel and fertilizer share 61.94% of the total consumption of energy, followed by electricity (16.01%), chemicals (10.19%), and human power energy (8.64%) (Hatirli et al., 2006). Pulse crops require lesser fertilizers than cereals, specifically nitrogenous fertilizers (~20 kg N ha^−1^), while cereals (rice and wheat) need 120–150 kg N ha^−1^. This saving of nitrogenous fertilizer by more than 100 kg ha^−1^ essentially saves energy (Figure 8). Amenumey and Capel (2014), in an experiment, reported that dry bean requires only 3% energy from fertilizers out of the total energy inputs that were significantly lower than those of oilseeds (10–41%), potato (25%), grain crops (19–60%), vegetables (12–30%), and fruit crops (2–23%).

### 8.1. Pulses for soil health restoration and chemistry in a rainfed agroecosystem

Approximately 30% of agricultural land, 20% of forest land, and 10% of rangelands are affected by land degradation (Ayub et al., 2019). In addition, nearly one-third of the soils on the planet have already been degraded, and over 90% are expected to be degraded by 2050 (IPBES, 2018). Globally, land degradation affects 2,000 million ha of land, where 1,500 million people reside (FAO, 2020a). Every year, 12 million ha of land is degraded throughout the globe, corresponding to 23 ha per min. On average, soil erosion caused a loss of 24 billion tons of fertile soil that threatens the livelihood of 1.5 billion people and is likely to displace 135 million by 2045 (United Nations, 2012). According to the aforementioned estimation, it could lead to up to a 50% loss in crop yield (FAO, 2020b). Several factors are responsible for land degradation; indiscriminate use of agrochemicals/poor quality water, mining, and deforestation are vital to them. During the green revolution in the 1960s, more cultivated areas were brought under a cereal-based cropping system that decreased the areas under pulses from 13.5 to 7.5 Mha in northern India, which drastically deteriorated soil quality, subsequently associated with lower fertility and caused a severe issue of sustainable crop production (Srivastava and Mukhopadhyay, 1997). These factors individually or collectively contribute to poor crop production by surging wind and water erosion, soil salinity, desertification, soil sickness, declining soil fertility, and heavy metal contaminations (Rani et al., 2019). To sustain the production system and ecosystem services to meet the global food demand with a sloping cultivated land, appropriate measures must be taken to stop further degradation and restore the already degraded soils. This helps to improve livelihood, boost food security, and aid people to adapt to the climatic extremes.

**Figure 9:**
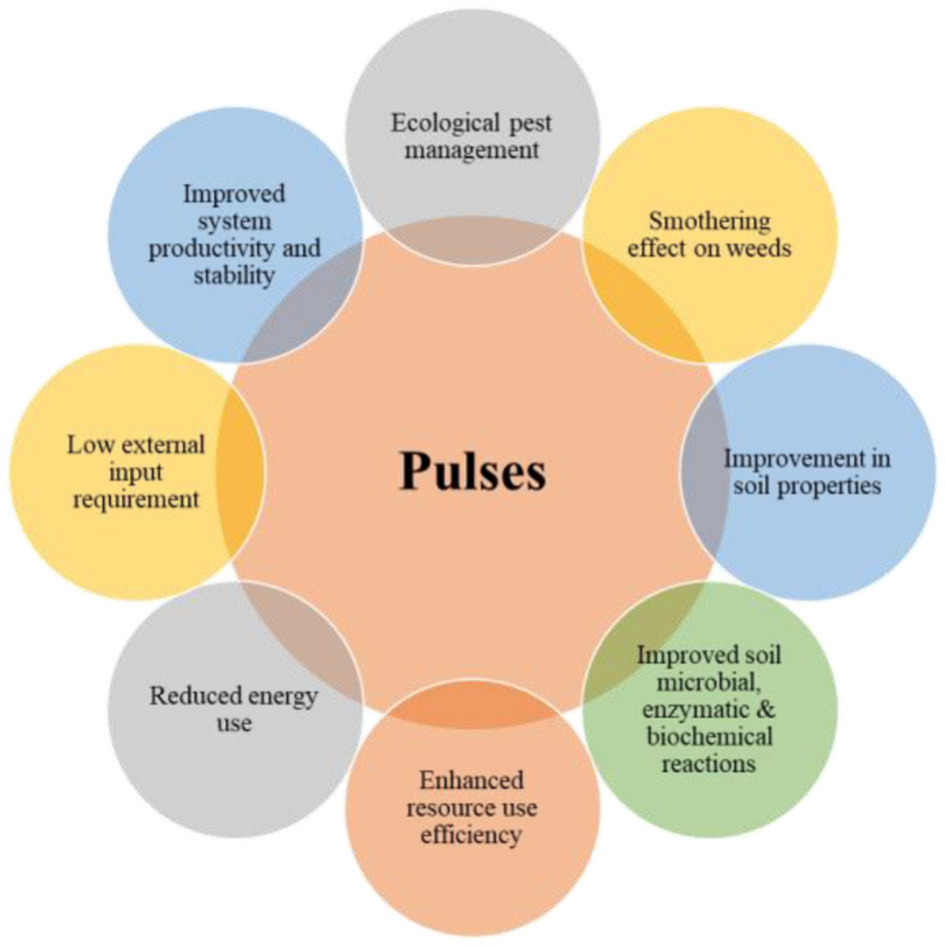
Pulses for soil health restoration and ecosystem services (Modified, Rani et al., 2019).

Pulse-based crop rotation increases crop yield, improves overall soil quality, and manifests sustainable production (Jangir et al., 2019). Improved fertility status, SOC, nutrient cycling, soil structure, and aggregation, enhanced soil microbial functions, and system productivity are important indicators to restore degraded soils (Figure 9). Pulses improve the fertility status of soil by enhancing SOC, available N, P, K, and other micronutrients of importance to plants. Compared to the crop residues of cereals and others, the residues of pulse crops are N-rich, along with a significant amount of C content. Soil bacteria take energy and N from these residues and facilitate a faster conversion of soil residues to SOC (Kumar et al., 2018). Likewise, short-duration pulses can help reduce C loss from fallow lands and boost C sequestration. Venkatesh et al. (2013) reported that soil under pulse production had an ~10% higher SOC stock for longer than that without pulses. The increment in SOC stock optimizes the sulfur (S) and N cycle in soil and compensates for other negative impacts of the environment on agroecosystems. The improved SOC tightly binds the soil particles to make the aggregates more stable and resistant to breakdown. These groups of crops increase the length of fungal hyphae in soil by 1.9 to 2.5 times (19–292 mg g^−1^) to that of soils without pulses. These fungal hyphae, in turn, prove to be a crucial binder of soil aggregates of >0.25 mm size, called macroaggregates, while the formation of micro-aggregates (<0.25 mm) takes place with the help of humic substances (Kumar et al., 2018). Thus, the enhanced soil aggregation promotes the formation of favorable soil structure and tilth. In Himachal Pradesh, India, Sharma et al. (2000) reported that a pulse-based system increased the percentage of macro-aggregates. Likewise, at Kanpur, India, Hazra et al. (2019) found an improvement in soil macroaggregates of >3.0 mm by 1.59, and 1.25 times in continuous pulse crops (35%) concerning uncultivated fallow (22%), and non-pulse crops (28%), respectively. They also observed a significant reduction in soil compaction cone index in continuous pulse crops (39) than in non-pulse crops (46) and uncultivated fallow (55) at 0.05% of the significance level. In Inceptisol (Typic Ustochrept) soils of Kanpur, Uttar Pradesh (India), Hazra et al. (2019) also reported higher retention of coarse macroaggregates (>0.25 mm) in pigeon pea–wheat and maize–wheat–mung bean rotations by 67.4 and 26.6% (*p* < 0.05), compared to the maize–wheat rotation, respectively. Similarly, the pulse-based cropping system also recorded significantly higher meso-aggregates (0.25–2.0 mm) over the maize–wheat system. Similarly, Kumar et al. (2019a) also found improved macro- and meso-aggregates, and aggregate mean weight diameter in legume-based crop rotations such as rice–chickpea–mung bean and rice–chickpea compared to the rice–wheat rotation in 0–20- and 20–40-cm soil depths. In addition to BNF, the nutrient-rich pulse residues add a considerable amount of N, P, K, and micronutrients to the soil ecosystem upon decomposition and mineralization that can be beneficially used by soil microbes and succeeding crops (Kumar et al., 2018).

The proliferation of soil microbes, enzymatic and associated biochemical processes into the rhizosphere regions due to root exudates adds to nutrient acquirement, soil nutrient reservoir, and retrieval of soil processes (Rani et al., 2019; Kumar et al., 2021). Hence, it reduces external chemical fertilization and advances ecological services and crop yield while minimizing the chemical pollution in soil, thus securing soil health. Pulses also ensure an enhanced growth of P-mobilizers and solubilizers, further advancing P acquisition and reducing external P fertilization. In addition, adaptive morphological and physiological approaches have been developed in pulses at multiple levels, including the continuation of higher P in root nodules, compared to surrounding tissues/organs, enhancement of root exudates' excretion, and root surface area. These approaches also include the articulation of transporters and aquaporins for improved P uptake and an increase in fixed N per unit of the nodule to reimburse for a decrease in the nodule number (Rani et al., 2019). Pulses promote the recycling of nutrients (i.e., C, N, and P), owing to their deeper, healthy, and profuse root network that helps in a more efficient resource utilization and reduces nutrient losses, *that is*, NO_3_ and thus enhances the nutrient recovery and NUE (Kumar et al., 2019b). These crops reduce NO_3_ leaching by 20–80% over non-legume crops by storing the inorganic soil N in the organic state between two crop seasons (Meisinger et al., 1991). In Denmark, Stagnari et al. (2017) found an increased N uptake by 23–59% in various field crops grown after field pea and lupin in rotation. In addition, the release of organic acids (e.g., piscidic acid and citric acid) into the soil through roots solubilizes the insoluble soil nutrients, including both macro- and -micronutrients, whose P solubility is well known. Long-term pulse cultivation significantly increases the buildup of these nutrients, which improves soil fertility status and, thus, soil health and quality.

### 8.2. Effect of pulses on nitrogen economy and biological fixation equations

**Figure 10:**
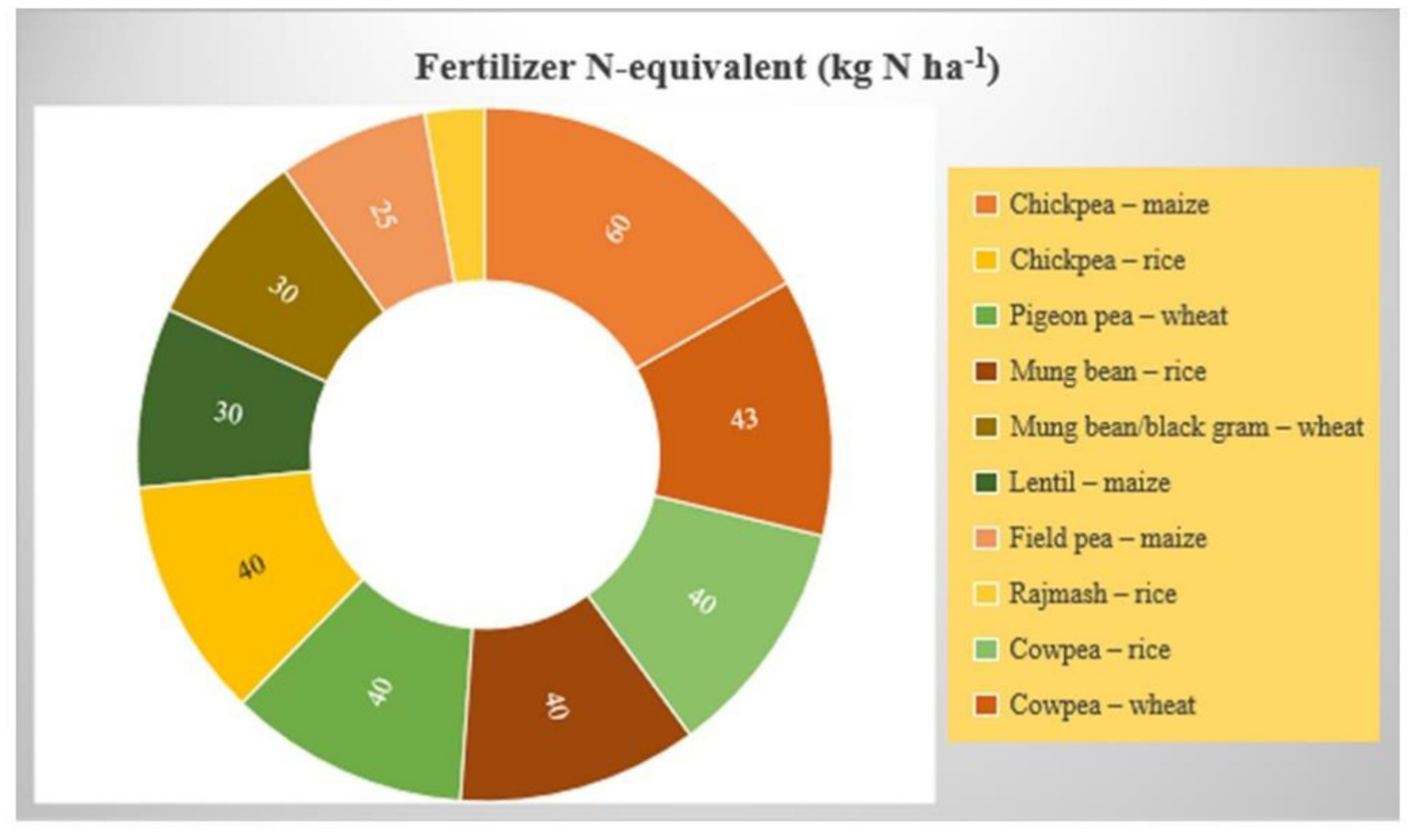
Nitrogen (N) economy due to the inclusion of pulses in the cereal-based system (*Data source*: Ali and Venkatesh, 2009).

It is well known that pulses leave a considerable amount of N in the soil after harvest. An estimate of the N economy in pulse-based cropping systems depicts the addition of 6,68,000 tons of N into the soil (Singh et al., 2009). Herridge et al. (2008) and Peoples et al. (2009) reported 47 and 87 Mt N year^−1^ through legume-based BNF and fertilizer application, respectively, that collectively cost over US$ 50 billion. The intrinsic N-fixing capability of pulse crops helps in meeting the N demands (N economy) of succeeding cereal crops due to its residual N effect (Figure 10). Mineralizable organic N, MBC, and mineral N bring about improvement in N economy. Furthermore, pulses are reported to have a high NO_3_ content in their soil profile, where the maximum increase in NO_3_ is observed with chickpea among *rabi* pulses with 20.4 kg ha^−1^. This extra NO_3_ is due to its reduced use during crop growth stages of pulses, which is termed the nitrate sparing effect.

The N-fixing ability takes place at the rate of 1.0 kg ha^−1^ day^−1^ in the growing season, which is considered the potential N-fixing ability of legumes within given environmental conditions (Kumar et al., 2017a). Approximately two-thirds of fixed N are available for the next growing season. On average, legumes can fix N around 30–150 kg N ha^−1^, which varies with rhizobial population, legume species grown, soil properties, management practices, and prevailing environmental conditions (Kumar et al., 2017a). A study in Australia showed an N economy of 40–90 kg N ha^−1^ in the first year, and the carryover effect was 20–35 kg N ha^−1^ through the inclusion of legumes in a cereal-based cropping system, in the second year (Peoples et al., 2015).

It is essential to assess BNF to evaluate the sustainability of pulse-based cropping systems. Quantifying BNF in the right way is crucial to achieving economic viability and long-term sustainability (Anglade et al., 2015). Different methodologies are used to estimate the N fixation of a crop, but no single method is universally accepted for all legume species. The most widely used methods are N differences, the Isotope dilution method (^15^N natural abundance and ^15^N enrichment method), and Acetylene reduction by nitrogenase enzyme and Xylem (Ureide) solute technique. Several empirical and simulation models are used recently to assess the predefined N fixation rate for different legume species (Liu et al., 2011). The principles and calculations of BNF using diverse methods are given in the following Table 10.

**Table 10:** Different methodologies and principles to assess biological *N*-fixation.

**S.No**.	**Method**	**Principle**	**Formula**	**References**
1.	*N* differences	The difference in total *N* accumulation in the shoot of the legume crop and control crop is determined as symbiotic *N* fixation	*Q* = [*N* yield (legume) – *N* yield (control)] + [*N* soil (legume) – *N* soil (control)]	Peoples et al. (1989)
2.	Nodule tissue	Total *N* plant α nodule tissue formed (weight)	Regression equation: *NF* = *b X*	Döbereiner (1966)
3.	*N* yields	Linear relationships with *N* yields	BNF (kg N ha^−1^year^−1^) $=\left[\alpha cult\times \frac{Y}{NHI}+\beta cult\right]\times BGN$	Anglade et al. (2015)
4.	**Isotope dilution method**			
a.	Natural ^15^N abundance	With increasing *N* fixation, there is a decline in the abundance of ^15^N in the N_2_-fixing plant as *N* assimilation from the soil is diluted by atmospheric *N* of lower ^15^N abundance fixed in root nodules of plant	$P=100\;\times \;\frac{\delta^{15}N\;\left(soil\;N\right)-\;\delta^{15}N\;\left(legume\;N\right)}{\left(\delta^{15}N\;\left(soil\;N\right)-B\right)}$	Peoples et al. (1989)
			$\%N=100\;\times \;\frac{{(}^{15}N_{ref})-\;{(}^{15}N_{leg})}{{(}^{15}N_{ref}-B)}\;$	Chalk and Craswell (2018)
b.	^15^N enrichment method		$P=100\;\times 1-\frac{\left(atoms\;{\%}^{15}N\;excess\;in\;legume\;N\right)}{\left(atoms\;{\%}^{15}N\;excess\;in\;soil\;derived\;N\right)}$	Peoples et al. (1989)
5.	Acetylene reduction by the enzyme nitrogenase	Detection of nitrogenase activity (as nitrogenase reduces acetylene to ethylene)	ARA = Nitrogenase activity × dry weight of the nodules	Chalk and Craswell (2018)
6.	Xylem (Ureide) solute technique	Differences in xylem *N* solute concentration between symbiotic plants and non-nodulated plants to assess their dependency on *N* fixation/soil mineral *N*	$Relative\;ureide\;index\;\left(\%\right)=\;\frac{\left(Ureide\;N\right)}{\left(Total\;sap\;N\right)}\;\times 100$	Peoples et al. (1989)
7.	Empirical models	Pre-defined *N* fixation rate α to harvested yield, plant *N* concentration and total *N* derived from *N* fixation	*Nfix = α · DM · f_*leg*_ · N_*con*_ · %Ndfa · (1 + R_*root*_)*	Liu et al. (2011)
8.	Simulation models	Pre-defined *N* fixation rate (*Nfix)* is estimated concerning response functions (soil/plant water status, soil temperature, soil/plant *N* concentration, *C* supply and growth stage of the crop)	*Nfix* = *Nfixpot fT fW fN fC f*g*ro*	Liu et al. (2011)

2^*^ NF, N fixed; b, regression slope of total plant N with nodule weight; X, corresponding nodule weight.

3^*^ α_cult_ and β_cult_, slope and intercept coefficients; Y, yield (kg N ha^−1^ year^−1^); NHI, N harvest index; BG, factor determining below-ground contributions, i.e., nodules, roots and rhizodeposition (factor 1.3 for pulses).

4^*^ P, percentage of pulse N fixed from atmospheric N; δ^15^N (soil N), obtained from non-N fixing plant grown in the same soil as a pulse; B, δ^15^N of N fixing plant grown with N as the sole source of N (values varies with different legumes); ^15^N_ref_, abundance of the ^15^N isotope in the reference plant; ^15^N_leg_, abundance of the ^15^N isotope in the pulse.

7^*^ DM, yield; f_leg_, legume proportion (if intercropped); N_con_, N concentration in pulse; %Ndfa, total N proportion derived from N fixation; R_root_, ratio of fixed N below and above ground.

8^*^ Nfixpot, N fixation rate (g N fixed day^−1^); fT – Soil temperature function, fW – Soil water status function; fN – Soil mineral N/root substrate N concentration function; fC – Substrate C concentration in plant/root function; fgro – Crop growth stage.

So, this group of pulses has a lot of benefits1 together with system productivity and overall economic efficiency of the crop production system.

## 9. Effect of pulses on system productivity and economic efficiency

With the increasing demand for food, declining per capita land area, and escalating human population, the improvement in crop productivity became necessary through sustainable intensification (SI) under a rainfed agroecosystem (Lal, 2017). In coming times, eliminating hunger and poverty and sustainable development will be major goals, and pulses could play a crucial role in achieving them, owing to their nutritious seeds, eco-friendly and less input requiring nature while increasing the carrying capacity of the land (Ali and Gupta, 2012). The SI of existing cropping systems, by involving pulses (e.g., intercropping/mixed cropping, relay cropping, sequential cropping, catch cropping, and ratoon cropping), improves the system productivity as a whole (Singh et al., 2009) and can feed the world.

Pulses benefit the cropping system by enhancing the pulse itself and subsequent crop yield and reducing input application because of improved soil health and fertility. Pulses can arrest the declining productivity of a continuous non-pulse-based system by improving the soil's physical, chemical, and biological environment (Singh et al., 2009). Thus, pulses being a part of an integrated plant nutrient supply chain of the cereal-based system should be promoted. There are several scientific reports on the role of pulses in improved productivity of subsequent cereal and oilseed crops while enhancing climate change resilience (Angus et al., 2015; Gan et al., 2015; Liu et al., 2019). For instance, pulses increase the yield of subsequent wheat crops by up to 35% in Northern America (Gan et al., 2015) and nearly 20% throughout the globe (Kirkegaard et al., 2008). The important indices used for assessing the productivity and economic efficiency of pulse-based cropping systems are Land Equivalent Ratio (LER), Crop Equivalent Yield (CEY), Relative Crowding Coefficient (RCC), Aggressivity Index (AI), Intercropping Advantage (IA), Competition Ratio (CR), Actual Yield Loss (AYL), Area Time Equivalent Ratio (ATER), Cropping Intensity (CI), Multiple Cropping Index (MCI), and Monetary Advantage Index (MAI) (Layek et al., 2018). In Van, Turkey, Ciftci and Ulker (2005) reported that the diversification of wheat (20%) through mixed cropping with lentils (80%) increased the LER to 1.15. The yield advantages of 20 and 15% were also obtained by diversifying mustard (*Brassica* spp.) through mixed cropping (75 + 25%) and intercropping (2 paired + lentil broadcasting), respectively, at Mymensingh, Bangladesh (Sekhon et al., 2007). The higher LER (1.61) was also found with lentil + linseed intercropping (3:2) in Bangladesh (Miah and Rahman, 1993). Kermah et al. (2017) reported higher productivity and economics of different grain–legume-based intercropping systems in the northern and southern Guinea savanna, respectively. The results indicated higher productivity and economic benefits in the intercropping system than sole cropping in terms of LER (1.16–1.81 and 1.07–1.54) in northern and southern Guinea savanna, respectively, due to an efficient and productive use of natural resources by intercropping. In Eastern Himalayan regions of India, Choudhary et al. (2014) reported a higher LER (1.63, 1.75, and 1.66) upon diversifying maize through intercropping with cowpea (1:2), french bean (1:2), and urd bean (1:2), respectively. By taking french bean as an intercrop in the upland rice field, the french bean increased the rice equivalent yield from 2.03 to 6.0 t ha^−1^ at midhills in Meghalaya and thus improved the total system's productivity per unit of land areas. Similarly, Oseni (2010), in the Bauchi in the northern Guinea savanna of Nigeria, reported higher RCC, CR, and aggressivity index while intercropping sorghum with cowpea in the different ratios over the sole cropping of both component crops. Furthermore, Kumar et al. (2015) highlighted the improved rice equivalent yield and system productivity by different pulse-based cropping systems. This observation shows a strong relationship between the crop yield/ N concentration in seed and the N fixed by pulse crops.

The impact of pulses on the productivity of pearl millet (*Pennisetum glaucum*)-based system is synergistic in the rainfed agroecosystem of northern India. Intercropping of mung bean with pearl millet increased equivalent yield of pearl millet grain by 15.3% over the sole pearl millet (Prasad and Nanwal, 2001). In another experiment, Varia and Sadhu (2011) observed the highest LER and RCC (3.47 and 2.04) in the pearl millet + mung bean intercropping system (1:2 and 1:1, respectively). While strip cropping of pearl millet + mung bean (4:4) produced 26% more yield benefits for the continuous pearl millet monoculture (Singh and Joshi, 1994). Likewise, strip cropping of pearl millet with mung bean (8:4) and cowpea (6:3) had higher LER, ATER, CR, MAI, RCC, and aggressivity, when compared to monocropping (Sharma and Singh, 2008). Aggressivity values showed that intercropping of mung bean and cowpea did not compete with pearl millet. The pearl millet + pigeon pea intercropping system also produced a greater pearl millet equivalent yield (PEY) (43.1 and 48.2 q ha^−1^) over the sole cropping of pearl millet and pigeon pea, respectively (Ansari et al., 2012). Pearl millet with moth bean (1:7) generated higher PEY, LER, and net return over sole cropping and other intercropping systems (Kuri et al., 2012).

Inclusion of peas in rotation with wheat positively impacts system yield, compared to continuous wheat. Superior performance of the pulse-based system over that of wheat monoculture was observed recently in terms of system yield, stability, and overall productivity (Liu et al., 2019). Farmers reported improved productivity of successive wheat crops (29.76 q ha^−1^) after pea, compared to continuous wheat (25.06 q ha^−1^). Among different crop rotations, the pea–wheat system showed a better performance in protein-based system yields that were 9–26, 22–82, and 26–82% higher than those of lentil–wheat, chickpea–wheat, and wheat monoculture, respectively (Liu et al., 2019). Similarly, Miller et al. (2003) observed that the wheat grain yield increased by 13 and 19% when grown as a subsequent crop after lentil and pea over wheat monoculture, respectively. This observation may be associated with peas' more profuse, shallow, and branched root system than chickpeas and lentils, which improved the nutrient and water uptake under stressed conditions (Liu et al., 2011). Due to the shallow root system, pea and lentil crops take water from top soils only and leave behind deep soil moisture for succeeding cereal crops, thereby improving crop performance in a rainfed agroecosystem (Liu et al., 2019).

The economic benefits of pulses in the agroecosystem are due to an improvement in total/system grain yields over the respective single crop. The saving of fertilizers' input, particularly N-fertilizer, irrigation, and higher returns from the inclusive pulse crop directly reflect the improved production economics. Competition among the component crops is an essential factor in the intercropping system that helps to know the compatibility of component crops; therefore, agroecosystems' economic and biological suitability and spatial arrangements can be determined. For example, intercropping of cowpea with maize had a higher actual intercropping advantage and yield loss over the respective monocultures (Takim, 2012). For instance, the pearl millet + mung bean intercropping system (30/60 cm) recorded the highest profitability in terms of net monetary returns (₹ 12,270 ha^−1^; US$ ~170) and benefit–cost (2.75) over sole cropping (Hooda and Khippal, 2000). Hooda et al. (2004), in another experiment, found that pearl millet + mung bean generated greater net monetary returns (₹ 16,043 ha^−1^; US$ ~220) and benefit–cost (1.90) over monocropping of pearl millet (₹ 13,120 ha^−1^; US$ ~180). Likewise, Singh et al. (2003) observed that mung bean + cluster bean (*Cyamopsis tetragonoloba*) intercropping in paired row planting was more profitable than other intercropping systems in terms of the cluster bean equivalent yield (1.77 t ha^−1^), net return (₹ 6,846 ha^−1^; US$ ~95), and income equivalent ratio (1.9). By looking toward the productivity gain and economic benefits of pulses in various ways, the identification, characterization, landmarking, and implementation of pulse-based agroecosystems in rainfed areas is essential.

## 10. Constraints, opportunities, and researchable issues for promoting pulses in a rainfed agroecosystem

In the era of the growing challenge of COVID-19, climate change, increasing food and protein demand of the global population, shrinking land and water resources, and rising prevalence of biotic and abiotic pressure, the production sustainability of the system is of prime concern. Existing non-legume-based cropping systems cannot maintain their long-term productivity level and are suffering from soil fertility depletion, extensive nutrient mining of natural nutrient reserves, declining productivity factor, reduced groundwater aquifer, decreasing soil C stock, augmented weed menace, and ecological instability (Kumar et al., 2017b). In this regard, pulses are in front most and have an immense potential for promoting ecosystem services, land restoration, resource conservation, and nutrient cycling while reducing groundwater extraction, chemical farming, and the ill effects of climate change.

There is a vast scope for increasing pulse production through the expansion of the horizontal area under pulses. The horizontal area can be increased by an inter-and/or mixed-cropping of pulses in crops such as sugarcane, potato, maize, cotton, mustard, pearl millet, and many other crops; by *utera/paira* cultivation in the rice-based system; and by the inclusion of pulses in an agroforestry system. In addition, the area can be increased significantly in huge rice fallows of India, Bangladesh, and Nepal by growing pulses after rice to take advantage of the residual soil moisture through agricultural conservation practices such as zero tillage and residue mulching. The production system can be intensified sustainably by replacing summer fallows with pulse crops such as mung bean, urd bean, and cowpea, which could bring additional 2–3 Mha areas under pulses in a more unsuitable rice–wheat system in Indo-Gangetic Plains (IGPs) of India (Kakraliya et al., 2018).

However, significant barriers to the successful adoption of pulses in rice and summer fallow are the timely unavailability of quality seeds of required varieties, lack of irrigation facilities, soil constraints, the poor financial status of farmers, and lack of clear vision of the government for sustainable intensification of these unused lands. In addition, a higher water requirement for summer pulses due to increased evaporative demands and a higher incidence of pests and diseases are also significant issues. Farmers of these regions must be supported with an efficient marketing network, enabling 100% procurement on minimum support price and technology transfer mechanisms, with particular emphasis on climate-resilient production technologies. The technology or management practices should be location-specific and economically flexible to understand better system ecology, obstructions, and possibilities for their higher adoption by the farmers. For the promotion of pulse cultivation, practical efforts have to be made through knowledge sharing, efficient technological intervention, site-specific input management, supplemental irrigation through a precise water applicator (*i.e.*, drip and sprinkler technology) coupled with rainwater storage, and the use of quality seeds of short-duration high-yielding stress-tolerant/resistance cultivars, according to the soil and environmental conditions.

Therefore, research must be initiated on following the problems of farmers by their active participation to advance the location-specific set of practices for easy adoption of technology. Yet, the potential of pulse crops for deep soil C sequestration remains poorly understood. An in-depth investigation is thus warranted to quantify the stabilization of pulse crops belowground root biomass. Increasing the content of the resistant C pool or humus is a vital strategy to enhance the SOC pool. A comprehensive assessment of cereal and legume residues in increasing the resistant C pool would help to develop strategic crop residue management. The potential of pulse genotypes for SOC restoration is another researchable issue. Variations in WUE and nutrient acquisition of some genotypes are also tested; however, there is scope to test these genotypes as to their C sequestration potential, so that the inclusion of pulses achieves SOC enrichment in the cereal-based system without incurring additional costs. Presently, the area under CA in the rice-wheat cropping system (RWCS) is increasing in South Asian countries, and pulses are vital for crop diversification. All the possible inclusions of pulses in the RWCS under CA practices need to be studied to advocate appropriate pulse-inclusive crop rotation for resource conservation and climate change mitigation. A comprehensive estimation of GHGs emissions in cereal–cereal and cereal–pulse systems provides useful information for the strategic management of GHGs emissions following the cropping system approach. In addition, investing in the shifts in soil microbial activities, abundance, and communities driven by different agricultural practices would be conducive to maintaining and enhancing the fertility and productivity of soils and protecting soil ecosystems against disturbances.

It is necessary to prioritize the formulation and implementation of novel policies by encouraging investment in research, technology transfer, farmers' training, knowledge sharing, capacity building, and application of advanced technologies based on the region-specific available resources. Global investments in pulses' research, development, and extension are too low (US$ 175 million) compared with cereals, *that is*, maize receives billions in investments. Therefore, investments in research and development (R&D) in pulses must be promoted among scientists and farming communities. Research on pulses is needed to increase productivity, improve climate resilience, and understand the health and nutritional benefits and value of ingredients and food processing. The Indian government is already working to promote pulse cultivation in rice fallows under the TRFA, NFSM-Pulses, and other smaller schemes/projects/programs. Its impact on increased pulse production has been seen in the last 3–4 years, but still, to make the program successful, more efforts, mainly centered on small and marginal farmers, are needed in this direction. Farmers can also be encouraged to cultivate pulses by providing financial assistance and subsidies or free accessibility of quality seeds to make programs and schemes more successful. The policy agenda needs to be centralized for improved productivity, risk management capacity, improved marketing facilities, financial assistance for technology adoption, and government subsidies and financial incentives. This calls for a holistic approach to develop an action plan by identifying the ecological and economic constraints and taking advantage of the existing opportunities by utilizing the available technical and scientific information. There is a need to bring together science and policies to generate a common platform that will be useful and applicable to all living entities, including soil. For achieving that purpose, a better coordination should be made possible among the scientific communities, farmers, and democrats for designing an efficient policy framework. With the help of scientists, farmers should also come forward to identify such management practices that are productive, eco-friendly, and have a wider scale adaptability. Research and development activities must be promoted in conservation practices and land-use decisions across the agroecological regions and most soil types. Farmers' feedback on the easiness, incentives, and economics of technology should be collected and accordingly, the gap has to be refined for further modification and improvement in the existing technology. Therefore, increasing the acreage and yield of pulses during the fallow period by adopting an appropriate package of management technologies may give birth to another green revolution and could prove to be a turning point in mitigating the food and nutritional security needs of millions of food-insecure and malnourished people.

In addition, still, some of the specific researchable issues have to be rectified to enhance the agronomic yield of pulses while sustaining the environment:

– Quantifying the rate of soil carbon sequestration and stabilization under a wide range of soils, agroecology, and pulses.
– Assessing GHGs emissions (specifically, N_2_O) and calculating the C footprint of diverse pulse-inclusive systems under a wide range of agroecology.
– Quantifying the N fixation capacity of different pulses and finding factors affecting them.
– Developing soil, climate, and system-specific set of practices to reduce N_2_O emission, dinitrogen–dinitrogen monoxide (N_3_O) leaching, and trade-off of BNF.
– Determining the impact of crop management strategies on the nutritional status of pulses, including the protein and micronutrient (Zn and Fe) levels.
– Adapting pulses to conservation agriculture (CA)-based systems and recognizing site-specific components of CA-based systems.
– Identifying agronomic management of soil and water to narrow the yield gap under resource constraints.
– Finding soil, water, and crop management systems for sustainable intensification of pulse-inclusive systems.
– Improving use efficiencies of different resources, including water, nutrients, energy, and land of the pulse-based system, under diverse soils and climates, and identifying the site-specific loopholes.

## 11. Conclusions

Pulses are healthy food, rich in dietary fiber, protein-energy, essential amino acids, vitamins, and minerals, and they have several other health benefits. Pulses are a precious source of nutritious food for 828 million insecure, 690 million undernourished, and 200 million malnourished people, mainly when the prediction showed the same hunger level in 2030 as in 2015. In addition to provisioning food, feed, and fuel, pulses have numerous subsidiary benefits for soil restoration, climate-resilient agroecosystems, and improved ecosystem services. Being hardy, they grow well in harsh environmental conditions, including rainfed agroecosystems where most of the food-insecure populations live. Most pulses are of short duration and fit well into an existing niche in most farming/cropping systems. Their natural ability to adapt to a wide range of soils and climates with limited additional resource requirements makes them the best crop for securing the food supply of global hunger under conditions of climate change. Pulses improve the yield of component/subsequent non-legume crops and their bonus yield, thus enhancing the overall system's productivity, economics, and environmental sustainability. Pulses aid in economizing N use in the system by adding 18–70 kg N ha^−1^ to the soil through the BNF process. Hence, they help to reduce the demand for chemical fertilizers by 25–30% and safeguard the soil and environment from the ill effects of chemical fertilizers. By cutting the demand for fertilizers and other inputs, the pulses save energy in the cropping system and thus reduce the C footprint. These crops effectively restore sick and degraded soil by improving the SOC stock and soil microbial functionaries and offsetting GHGs emissions in a rainfed agroecosystem. Subsequently, pulses in the production system enhance adaptation to climate change by conserving/maintaining soil moisture, SOC, and health. Overall, pulses promote the four pillars of sustainability, *that is*, economic, environmental, social, and institutional, through diverse interactive natural progressions. Pulses also directly contribute to the UN's SDGs for human wellbeing and natural resource conservation. Thus, considering the future plans to cater to the feeding needs of the exploding population and the significance of pulses in health, nutrition, and ecosystem management, the areas covered under the production of pulses must be increased to 296 Mha by 2030 and 238 Mha by 2,100, in addition to a productivity of 1,800 and 2,150 kg ha^−1^, respectively.

## Author contributions

Conceptualization and validation: SK, KG, and RM. Data curation: SK, SS, and RM. Formal analysis: SK and SB. Methodology: SK, RM, SB, and SS. Resources: RM and RJ. Supervision: KG, CS, and RM. Visualization: SK, SS, RM, and SB. Writing—original draft: SK, SS, RM, SB, CJ, and KM. Writing—review and editing: RM, SK, CS, KG, RJ, and CP. All authors contributed to the article and approved the submitted version.
